# Massively parallel characterization of engineered transcript isoforms using direct RNA sequencing

**DOI:** 10.1038/s41467-022-28074-5

**Published:** 2022-01-21

**Authors:** Matthew J. Tarnowski, Thomas E. Gorochowski

**Affiliations:** 1 School of Biological Sciences, University of Bristol, Tyndall Avenue, Bristol, BS8 1TQ UK; 2 BrisSynBio, University of Bristol, Tyndall Avenue, Bristol, BS8 1TQ UK

**Keywords:** RNA sequencing, Synthetic biology, Expression systems

## Abstract

Transcriptional terminators signal where transcribing RNA polymerases (RNAPs) should halt and disassociate from DNA. However, because termination is stochastic, two different forms of transcript could be produced: one ending at the terminator and the other reading through. An ability to control the abundance of these transcript isoforms would offer bioengineers a mechanism to regulate multi-gene constructs at the level of transcription. Here, we explore this possibility by repurposing terminators as ‘transcriptional valves’ that can tune the proportion of RNAP read-through. Using one-pot combinatorial DNA assembly, we iteratively construct 1780 transcriptional valves for T7 RNAP and show how nanopore-based direct RNA sequencing (dRNA-seq) can be used to characterize entire libraries of valves simultaneously at a nucleotide resolution in vitro and unravel genetic design principles to tune and insulate termination. Finally, we engineer valves for multiplexed regulation of CRISPR guide RNAs. This work provides new avenues for controlling transcription and demonstrates the benefits of long-read sequencing for exploring complex sequence-function landscapes.

## Abstract (web summary)

Transcriptional terminators are generally viewed as hard endpoints for transcribing RNA polymerases. Here, the authors reimagine terminators as transcriptional valves with predictable read through. They engineer and characterize 1780 valves and use them for multiplexed gene regulation.

## Introduction

The ability to precisely control when and where genes are expressed is crucial for engineering the behavior of living cells. To achieve this, bioengineers have predominantly focused on developing genetic parts to regulate transcription and translation initiation rates for genes of interest^1^. However, endogenous gene regulation is often multifaceted, employing diverse mechanisms that affect the stability and processing of DNA, RNA, and proteins to create complex regulatory programs^1,2^.

**Fig. 1:**
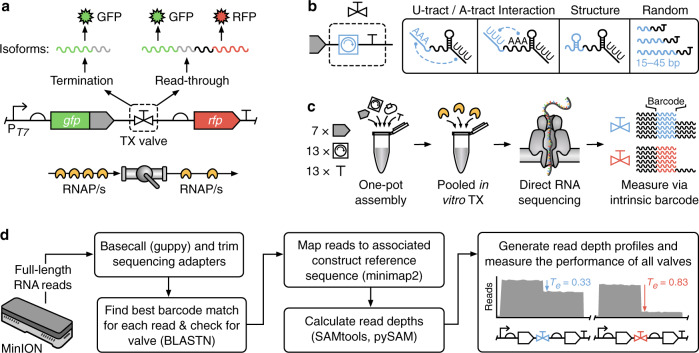
Massively parallel characterization of transcript isoforms using nanopore-based direct RNA sequencing. **a** Schematic of the genetic construct used to characterize transcriptional valves. A transcriptional valve controls the ratio of RNA polymerase (RNAP) termination to read-through and thus the proportions of transcript isoforms produced. **b** Our modular transcriptional valves comprise a ‘core terminator’ and ‘modifier’ sequence (blue) used to tune termination efficiency. Various modifiers were considered to interact with the U- and A-tract of the core terminator, form small secondary structures in the RNA, and act as different length inert spacing elements. **c** The steps involved in the assembly of the modular transcriptional valve library and its pooled characterization using nanopore-based direct RNA sequencing. **d** Analysis pipeline used to generate valve-specific read depth profiles and calculate termination efficiencies (*T*_e_) from pooled direct RNA sequencing data. Key computational tools are shown in parentheses.

Transcript isoforms are commonly used by eukaryotes to diversify the RNA products produced by a single gene through the subsequent processing of a transcript by splicing machinery^3^. Although such machinery is not generally present in prokaryotes, there is a growing realization that these organisms also generate transcript isoforms by utilizing incomplete transcriptional termination^4–6^. In this case, two transcript isoforms are possible: one ending at the terminator (if termination succeeds), and the other reading through (if termination fails) (Fig. 1a).

The design of synthetic terminators often focuses on increasing their termination efficiency^7^ as a way of insulating multiple genetic parts and devices from each other^8^ and the host genome^9^. Engineering terminators with a variety of efficiencies, insulated from an upstream sequence, or tuned for manipulating transcript isoform ratios^10^ has yet to be widely explored. However, an ability to design transcript isoforms in this way could open new avenues to control the stoichiometry of multi-gene expression purely at the level of transcription. Furthermore, this regulatory approach could be more efficient and impose less burden^11–13^ than other more commonly used methods, like operons, as not all RNA polymerases (RNAPs) would need to synthesize full-length multi-gene transcripts and thus could be freed more quickly for other tasks. This approach would also be suitable for differential expression of purely RNA-based regulators (e.g., small RNA triggers for toehold switches^14^ or gRNA arrays^15^) where translation into protein does not occur.

A major bottleneck when developing new genetic parts is the time and effort needed to characterize libraries of parts to understand their design principles and build predictive models of their function. For transcriptional terminators, a commonly used approach in vivo is a fluorescence assay in which two different fluorescent reporter proteins have the terminator to be tested placed between them. By comparing the ratio of fluorescence for each reporter with and without the terminator present, it is possible to indirectly quantify the fraction of transcriptional read through and the termination efficiency of the terminator^7,16,17^. More recently, methods employing RNA sequencing (RNA-seq) have been used to provide a more detailed and direct measurement of termination at a nucleotide resolution by allowing for transcriptional profiles capturing RNAP flux along the DNA to be inferred. Drops in RNAP flux within these profiles can be used to measure both termination efficiencies as well as the precise location at which these events occur^4,18,19^.

A challenge with all in vivo approaches is that they often need to make assumptions about the stability of the cellular environment and properties of the transcripts and proteins produced. However, these may not always hold: variations in mRNA stability^20^, the occlusion of adjacent ribosome binding sites due to terminator mRNA secondary structures, the impact of a terminator on the translational coupling of neighboring genes^7^, and transcription-translation coupling^17^ could all potentially play a role and affect the accuracy of the measurements made. Such differences may explain why in vitro and in vivo termination efficiencies have not been found to correlate well^21^. Nonetheless, insights into in vitro termination have proven useful. For example, in vitro assays using chip-based capillary electrophoresis^22^ have been used to identify terminators which have subsequently been used both in vitro^23^ and in vivo^24^. A bigger issue facing all of these approaches though is their limited throughput and scalability, with designs often needing to be tested separately. This severely hampers the exploration of the vast genetic design space and limits our ability to understand how terminators are effectively engineered.

In this work, we aim to address these issues and develop new genetic parts for regulating multi-gene constructs. Rather than treating terminators as the endpoint of a transcript, we consider them as “valves” able to regulate the RNAP flux passing through a point in DNA and thus the ratio of transcript isoforms that occur (Fig. 1a). To explore this approach, we iteratively design several large libraries of modular transcriptional valves for T7 RNAP. The use of T7 RNAP ensures that the parts developed can be used in both cell-free systems and broadly across organisms in the future. We show how nanopore-based direct RNA sequencing (dRNA-seq) can be used to characterize the in vitro function of entire pooled libraries at a nucleotide resolution. Using this data, we are able to infer and test design principles and show how genetic context can be used to tune termination efficiency or insulate a terminator’s performance from local variable sequences. Finally, we show how engineered valves can be used to regulate an array of CRISPR guide RNAs. Our methodology and experimental findings offer a means to control RNAP flux and transcript isoforms in genetic circuits and demonstrate how long-read sequencing can improve our understanding of large genetic design spaces.

## Results

### Designing transcriptional valves to control transcript isoforms

To demonstrate how transcriptional valves might be built, we attempted to construct proof-of-concept designs for T7 RNAP. T7 RNAP was selected due to its broad use in synthetic biology, which stems from the fact that it is a single-subunit RNAP with high processivity, making it ideal for both in vitro use^25^ as well as an orthogonal transcription system in vivo^26,27^. While diverse terminators are available for the native *E. coli* RNAP^7,16^, for T7 RNAP only a single terminator exists in the T7 phage genome^28^ and only a few alternatives have been characterized^22,29,30^. Furthermore, while termination of RNAP in model microorganisms like *E. coli* and *S. cerevisiae* has been extensively studied^31^, T7 RNAP termination has many unknowns such as alternative intrinsic terminators beyond those in the T7 phage genome and the bidirectionality of termination.

Specific features of a terminator, such as its hairpin structure and U-tract, can strongly influence termination efficiency^7,32^. Therefore, as a basis for an initial library of transcriptional valves, we chose 13 different intrinsic terminators to act as “core terminator” elements (T). We began by selecting the single terminator from the T7 phage genome (T27)^28^, which has previously been characterized in vitro^22^. To test its possible bidirectionality, a feature that terminators for other RNAPs have been shown to exhibit^7^, it was included in a reverse orientation in our designs^22^. Beyond the native T7 phage terminator, *E. coli* terminators present another source of these parts and have been shown to terminate T7 RNAP in vitro^22,33^. Therefore, 11 intrinsic rho-independent terminators were selected from the *E. coli* genome spanning a wide range of termination efficiencies in vivo^7^. Finally, a negative control terminator (T33) was designed. This consisted of a random non-coding sequence generated by R2oDNA designer^34^ and was further verified to not contain a strong hairpin in the mRNA secondary structure using the Vienna RNAfold tool^35^.

The genetic sequence immediately upstream of a terminator-hairpin also influences termination^16,17^ and we reasoned that this region could be used to fine-tune the termination efficiency of a valve. We, therefore, included a “modifier” part (M) in our transcriptional valve design and developed 13 different modifier sequences (Fig. 1b). Our first set of modifiers contained motifs designed to interact with canonical regions of a terminator hairpin sequence. Specifically, modifiers M10 and M11 were designed to interact with possible U- and A-tracts within a terminator by containing complementary homopolymers of adenine or uracil, respectively^7^. A further modifier M13 was designed to encode a small RNA secondary structure with the goal of affecting RNA structure formation near the terminator part. Beyond tuning termination efficiency with RNA interactions and structures, it has been shown that inert random sequences can play an insulating role, improving the robustness of a genetic part’s performance when used in different genetic contexts^17,36^. It is also known that the upstream genetic context of intrinsic terminators influences termination in a distant-dependent manner^16,17^. Thus, we decided to include a selection of modifiers of different lengths (M13–M16: 15 bp, M17–M19: 30 bp, and M20–M22: 45 bp) where each was a random non-coding sequence generated by R2oDNA designer^34^.

To assess the robustness of each valves’ termination efficiency to local upstream genetic context, our library also included “spacer” elements (S) (Fig. 1b). These did not form part of the transcriptional valve, but instead allowed us to see how a particular valve might behave when used in combination with other components (e.g., coding regions). Using the NullSeq tool^37^, we generated 7 random and genetically diverse 33 bp long spacers with a nucleotide composition similar to coding regions of *E. coli* that could be placed at the 5′ end of a valve. Each spacer had a stop codon “TAA” at its 3′-end, though this was not utilized in our in vitro transcription assay. Taken together, our spacers, modifiers, and core terminators could be combinatorially assembled to create a total of 1183 unique designs able to regulate RNAP flux and provide valuable information regarding the design principles of transcriptional valves.

### Combinatorial assembly of a transcriptional valve library

A one-pot pooled combinatorial DNA assembly method was used to physically construct the final set of transcriptional valve designs (Fig. 1c, see “Methods” section)^38^. *E. coli* cells were transformed with this pooled library and ~500,000 colonies (>400-fold library coverage) were selected from plates via scraping before their pooled DNA was extracted. Such a high fold-coverage ensured representation of each design in the sample (Supplementary Note 1)^39^.

Nanopore-based long-read DNA sequencing (DNA-seq) was then used to verify the successful combinatorial assembly of every design. This showed that all designs were present with an even distribution of parts but an uneven distribution of designs (Supplementary Fig. 1). Part frequencies matched the ratios expected from the equiprobable assembly, except for short parts 15 bp long which were under-represented. This may be due to reduced assembly efficiency for shorter parts. These part frequencies were used to predict design frequencies, of which 91 had >20% absolute deviation between predicted and measured frequency. Certain parts were overrepresented in these designs (M14: 3-fold and T18: 2.3-fold) indicating that the abundance of constituent parts did not solely dictate design abundance within the library.

Finally, we investigated DNA assembly fidelity by generating accurate consensus sequences from the long-read DNA-seq data (see “Methods” section). Comparing the reference and consensus sequence for each design we found a mean of 0.6 single nucleotide polymorphisms (SNPs) per design, with 40% of designs having no SNPs (Supplementary Fig. 1).

### Pooled characterization using direct RNA sequencing

Existing fluorescence^7^ and sequencing-based^18,19,40^ methods for measuring transcriptional termination are ill-suited to characterizing a large pooled library of genetic parts at nucleotide resolution. Therefore, we used nanopore-based direct RNA sequencing (dRNA-seq) to provide full-length reads of transcript sequences^41^. Crucially, each transcript isoform encodes its associated transcriptional value sequence either at the 3′-end, if termination was successful, or within the body of the transcript, if transcriptional read-through had occurred—the design’s sequence, therefore, acts as an ‘intrinsic barcode’ that is present in every read. This allowed for individual reads to be attributed to a particular design without the need to separate and barcode them before preparing the sequencing library.

Such an approach is not possible when using more common short-read RNA-seq because the transcriptional valve sequence for reads generated by read-through events will not always be located near the transcript end, and so would not be captured by the short read length if sequenced using normal approaches. Targeted short read sequencing could potentially be used to overcome this issue but would suffer from biases present during the reverse transcription (RT) step and subsequent PCR amplification^42–44^. Long-read dRNA-seq allows for the whole library to be directly assayed as a single pooled sample, without the need for RT, PCR or any assembly, and the data demultiplexed to simultaneously produce separate read depth profiles for each design. Finally, by comparing the ratio of transcript isoforms for each design (i.e., read depth directly before and after the transcriptional valve) a termination efficiency can be calculated (Fig. 1c).

While this approach removes the need to separate and attach unique ‘barcode’ sequences to each design when characterizing the library, it does rely on each transcriptional valve having a sufficiently different sequence for each read to be accurately mapped to a single design. Read accuracy for nanopore-based dRNA-seq is, at present, lower than for standard Illumina-based short-read RNA-seq (median read accuracy of 80–90% versus >99.9%, respectively^45^) and although this gap is closing with improvements to basecallers and sequencing chemistries, analysis pipelines need to be carefully tuned and validated to ensure accurate demultiplexing of reads when dRNA-seq is used in this way.

### Optimizing the computational analysis pipeline

To optimize the analysis pipeline and ensure that our library could be accurately characterized, we developed a simple computational model to simulate the error-ridden reads that would be obtained after dRNA-seq. In our simulations, errors took the form of random nucleotide substitutions that occurred at a 15% substitution frequency. While other types of error such as insertions, deletions and elevated error rates at homopolymers were not included in our model, we found that our simulations were able to identify key parameters and criteria for designing parts with a sufficient dissimilarity for effective demultiplexing.

To demultiplex the dRNA-seq reads, the BLASTN tool was used to find all possible alignments between a read and the library of designs, with the best matching design being chosen^46,47^. Optimizing the BLASTN parameters is crucial for accurate characterization and so computational analyses were performed where a smaller library (540 designs, Supplementary Fig. 2) was used to systematically explore the role of each BLASTN parameter. Each design was given a random termination efficiency and had a set of full-length terminated and non-terminated reads generated based on our model. These reads were then pooled for all the designs and attempts made to demultiplex and infer the original termination efficiencies for each design. This allowed us to generate an optimized set of parameters that allows each design to be accurately identified (see “Methods” section).

The final computational demultiplexing and analysis pipeline (Fig. 1d) involved aligning the sequences of all designs against all reads using BLASTN with optimized parameters and then associating each read with the design that had the best alignment score (see “Methods” section). Reads for each design were then mapped to the appropriate reference sequence and design-specific read depth profiles generated, filtering out any reads where no terminator was present after alignment and mapping. Finally, termination efficiencies were calculated for each read depth profile as *T*_e_ = [*R*(*x*_s_*)* − *R*(*x*_e_)]/*R*(*x*_s_*)*, where *R*(*x*) is the read depth at position *x* in the genetic construct, and *x*_s_ and *x*_e_ are the start and end nucleotide position of the transcriptional valve, respectively.

### Characterizing transcription termination at nucleotide resolution

In vitro transcription using T7 RNA polymerase of the entire pool of DNA constructs followed by dRNA-seq enabled us to rapidly assay the performance of each design simultaneously. To ensure the accuracy of our measurements, a detailed analysis of the generated read depth profiles was performed, which revealed several key features in line with other dRNA-seq studies (Supplementary Note 2)^48^. We also developed a mathematical model that allowed us to correct for unwanted deviations between actual and measured *T*_e_ (Supplementary Note 3; Supplementary Figs. 3–6). We found that transcript abundances were weakly correlated with DNA construct frequencies (*R*^2^ = 0.22), with strong terminators over-represented in the dRNA-seq data. A good reproducibility was observed for *T*_e_ values between replicates (*R*^2^ = 0.99) and for terminators shared across separately assembled libraries with different part compositions (Supplementary Fig. 7). This resulted in 98% of designs having a difference of <5% in *T*_e_ across the experimental replicates of our initial library.

**Fig. 2:**
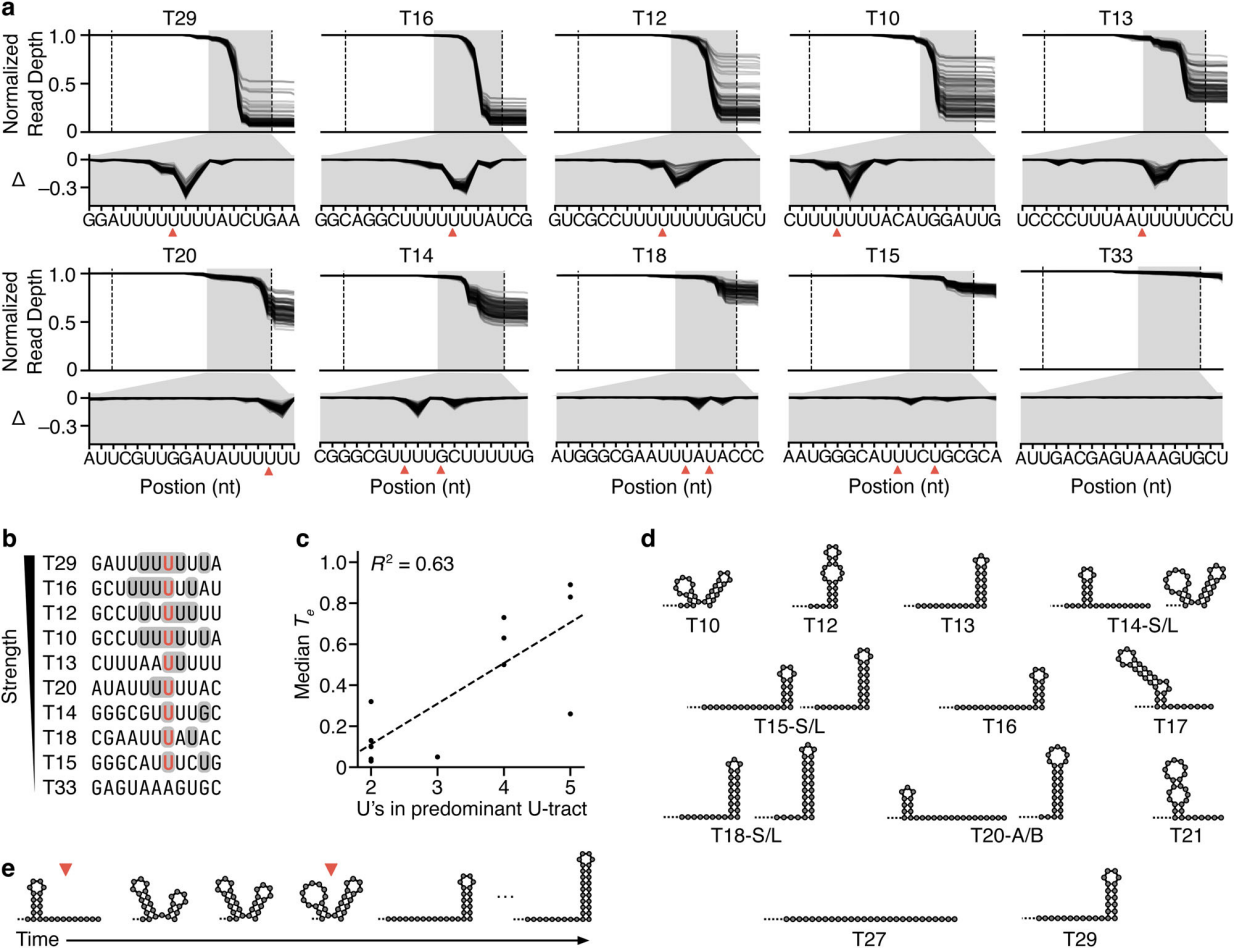
Nucleotide resolution read depth profiles reveal terminator phenotypes. **a** Normalized dRNA-seq read depth profiles for functioning terminators and non-terminator control (T33). Each line corresponds to a design. Dotted lines denote the start and end of the core terminator. Red triangle indicates the final nucleotide of the dominant point(s) of termination. The gray-shaded region is expanded in the lower panel showing the change in normalized read depth at each nucleotide position with respect to the previous nucleotide (Δ). **b** Termination locations for functioning terminators. Many terminators terminate at more than one position and these points are indicated with shading. The most common point of termination is colored red. **c** Median termination efficiency (*T*_e_) for designs containing each core terminator compared to the number of U residues in the U-tract, which is the 8 nt sequence upstream of the point of termination. *R*^2^ is the square of the Pearson correlation coefficient. **d** Secondary structure predictions of transcripts using a co-transcriptional folding simulation at the measured point of termination for each terminator (for inactive terminators, the sequence downstream of the hairpin with a maximal number of U residues was used). Two structures are shown for T20; T20-A is formed first, and T20-B lies over a large energy threshold. For weak terminators, the structure prediction for both the shorter (S) and the longer (L) transcripts produced are shown. **e** Co-transcriptional RNA secondary structures predicted for T14 as it is transcribed, with dominant points of termination indicated. Source data are provided as a Source Data file.

A valuable feature of part characterization by RNA-seq is the ability to extract nucleotide resolution insights from the read depth profiles. To enable comparisons between our designs where total numbers of reads for each varied, we generated profiles normalized by the read depth at the start of the transcriptional valve such that drops due to termination corresponded to a fractional change (Fig. 2a). We also calculated Δ-values corresponding to the change in normalized read depth at each nucleotide position with respect to the previous nucleotide, enabling us to pinpoint and compare changes more easily. We found that the maximum Δ-value for each design was proportional to its *T*_e_ and amounted to approximately 40% of the total *T*_e_ value. Each terminator maintained a predominant termination pattern (as shown by its Δ-profile), which varied in amplitude depending on the upstream modifier and spacer (Fig. 2a). The ability to observe these nucleotide resolution changes demonstrates a further benefit of the pooled dRNA-seq over more commonly used methods based on fluorescent reporter proteins.

The termination pattern is an important phenotype and we found that termination does not occur at a single nucleotide location; for each of our core terminators, it occurred over several nucleotides. While the *T*_e_ of a terminator did vary across genetic contexts, in general, the termination pattern remained consistent. These patterns revealed that termination often fluctuates nucleotide by nucleotide, resulting in multiple drops in the Δ-profiles and therefore multiple transcript isoforms.

As expected, drops in read depth for each valve occurred within the corresponding U-tract (Fig. 2b). We found that termination was possible with as few as 2 U’s, but that maximum drops in the profiles occurred at a similar point (after 4 or 5 U’s) for the stronger core-terminators. This likely captures a position where a combination of optimal T7 RNAP pausing and weakened stability of the transcription elongation complex leads to the formation of the core terminator hairpin sufficient to effectively facilitate termination. The number of U’s in the U-tract at the point of maximal termination showed a correlation with *T*_e_, although there were some outliers (T20, T14; Fig. 2c). This matches a previous finding for *E. coli* RNAP termination^7^. However, termination was found to always reach a peak with a U-tract that comprises fewer than the maximum possible number of U’s in the U-tract.

Using this data, it was possible to predict RNA secondary structures at the various points of termination and assess their potential influence. To do this we simulated co-transcriptional folding^49^ of the terminated sequence at the point of maximal termination (Fig. 2d), assuming a previously reported transcription rate of 333 nt/s^50^ (see “Methods” section). We removed the final 8 nt, which have been shown to base-pair with the DNA template in the T7 RNAP transcription elongation complex^51^, and studied the structure with the lowest folding energy. The strongest terminators were predicted to form terminating hairpins at their 3′-end, proximal to the elongating T7 RNAP. On the contrary, T20 was found to get locked in a secondary structure involving a hairpin ending 16 nt upstream of the T7 RNAP, meaning that it remained a weak terminator despite having a long U-tract. To reach an effective terminating hairpin T20 would have to surpass a large energy barrier.

There were three inactive terminators (T17, T21, T27) and the negative control (T33). Each of these could have a maximum of 4 U’s in the U-tract and none were predicted to form a hairpin proximal to the U-tract, which is likely the cause of their inactivity. This showed that T27, the reverse oriented phage terminator, was not able to efficiently terminate T7 RNAP bidirectionally. The three weakest active terminators (T14, T18, and T15) all had only 2 U’s in their U-tract of their most common transcript isoform, saw termination at multiple separated points, and were predicted to form a hairpin proximal to the U-tract (Fig. 2d). It is not clear why T14 gives stronger termination than T15 and T18 though one element that might increase *T*_e_ is the unique double hairpin structure that can form in the core terminator at the later points of termination. The second peak in termination for T14 coincides with the last point at which this double hairpin is predicted to exist (Fig. 2e). T15 and T18 on the other hand are predicted to form a single long hairpin structure.

An ability to engineer the precise point(s) of termination and therefore dominant 3′-UTR sequences may be important in deciding gene stoichiometries in vivo as it could potentially affect mRNA degradation rates, however, this is contested^52,53^. To assess this, we reviewed the possible transcripts produced by the characterized terminators and this showed that the final nucleotide of transcript variants can be either A, C, U or G. Frequently the dominant transcript terminated within a stretch of U’s though less frequently observed transcripts were found to terminate immediately after a stretch of U’s. Further investigation revealed that in some cases, upstream sequence could tune the major point of termination. For example, modifiers M10, M11, M12, and M15 showed different stoichiometries of two types of transcripts produced by T13.

We also undertook an analysis of other general biophysical parameters that may play a role in termination (e.g., GC content and minimum free folding energy). However, none of these were correlated with measured *T*_e_ (Supplementary Fig. 8).

### General termination properties of the initial valve library

**Fig. 3:**
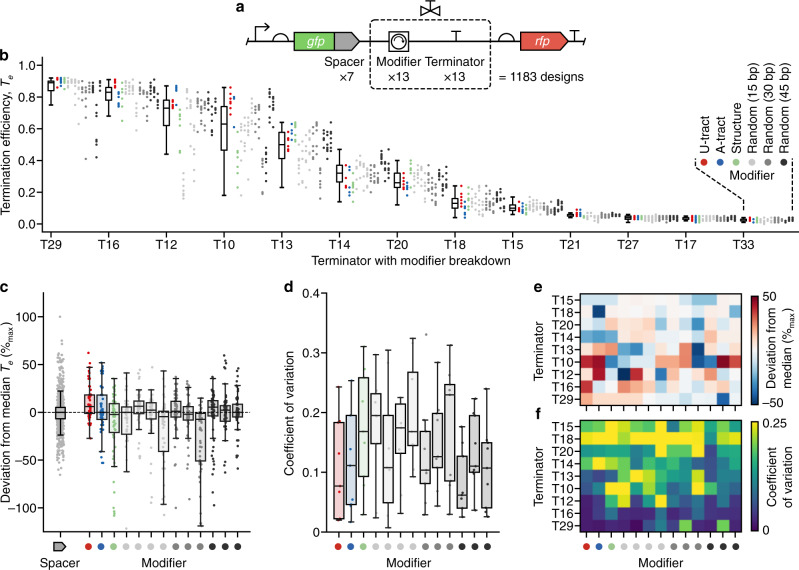
Characterization of a T7 RNA polymerase transcriptional valve library. **a** Structure of the transcriptional valve library. Gray ‘spacer’ elements fused to *gfp* are 33 bp random sequences used to assess the sensitivity of a transcriptional valve to nearby sequence context (full sequences available in Supplementary Data 1). **b** Termination efficiency (*T*_e_) for designs in the library (Supplementary Data 2). Each point denotes the *T*_e_ value for a unique genetic construct color-coded by the modifier present. Points are grouped by core terminator with a box plot summarizing the data for all associated constructs. *n* = 91 for each core terminator, apart from T13 where *n* = 90 and T18 where *n* = 89. **c** Percentage deviation in *T*_e_ (as a percentage of maximum possible deviation) for each construct from the median of all constructs containing the same core terminator. Each point corresponds to a single construct and points are grouped by modifier. Only data for active terminators (median *T*_e_ > 0.05; T29, T16, T12, T10, T13, T14, T20, T18, T15) are shown. *n* = 819 for spacers and *n* = 63 for all modifiers, apart from M14 where *n* = 60. **d** Coefficient of variation (CV) of *T*_e_ values across spacer variants for active terminators grouped by modifier. *n* = 9 for each modifier. **e** Terminator and modifier breakdown of the CV of *T*_e_ values across spacer variants for active terminators. **f** Terminator and modifier breakdown of the percentage deviation in *T*_e_ (as a percentage of maximum possible deviation) for active terminators. Random sequence modifier parts in all plots: (left–right) M10–M22. All box plots show the median as their center value, the bounds of the box are the interquartile range (IQR; 25–75%), and whiskers are from Q1 – 1.5 × IQR to Q3 + 1.5 × IQR, where Q1 and Q3 are the 25% and 75% quartile, respectively. Source data are provided as a Source Data file.

Overall, *T*_e_ varied from 0 to 0.94 across the library with core terminators displaying varying levels of *T*_e_ and sensitivity to different modifier and spacer parts (Fig. 3). Grouping designs by their core terminator showed that each had a unique median *T*_e_ and variability that differed between terminators (Fig. 3b). We found that valves containing the non-terminator part (T33), reverse oriented T7 phage terminator (T27) and two of the *E. coli* terminators (T21 and T17) showed little to no termination (*T*_e_ < 0.05). The remaining nine *E. coli* terminators displayed a range of termination efficiencies for T7 RNAP with median *T*_e_ varying from 0.01 to 0.91, which was heavily influenced by upstream sequence context. The variety of *T*_e_ values observed would allow for a wide range of transcript isoform stoichiometries to be produced from 1:1 to 11:1. However, we were interested to know if patterns within this data might offer insight into the capacity of each terminator to be tuned or insulated. For example, valves displaying a wide range of *T*_e_ values for the same core terminator would indicate that the terminator is highly tunable, while a small range of *T*_e_ for a valve used with differing spacer elements would suggest that it is able to insulate its function from upstream sequence context.

### Tuning the strength of transcriptional valves

It is known that local sequence context can also be used to effectively alter the function of many types of genetic part^8,54–56^. We, therefore, designed modifiers in our initial library with the aim of being able to tune the strength of a valve. Analysis of the characterization data showed that changes in upstream genetic context (both spacer and particularly modifier sequences) could significantly influence termination strength, allowing *T*_e_ to be varied over a range of up to 0.68. The ability to tune each core terminator varied, with the *T*_e_ of T10 being most tunable and T16 being the least. The capacity to tune terminator strength could arise from the diversity of co-transcriptional structures that form proximal to the U-tract when interacting with the modifier (Fig. 2d). Therefore, to create a library of highly tuned transcriptional valves, it is important to ensure the core terminators are themselves tunable.

Large variability in the magnitude of tuning was seen across the different valves we tested suggesting that sequence-specific features play a key role in modulating the precise termination efficiency. Spacers were found to not have such a systematic effect. Nonetheless, some valves were highly influenced by spacers, emphasizing the importance of upstream sequence in the region up to 120 nt upstream of the point of termination. For designs grouped by spacer, the median percentage deviation from the median *T*_e_ of the valve they contained was found to be less than 5% (Fig. 3c), suggesting that tuning of termination efficiency is best achieved by varying sequence context close to the core terminator part.

In general, each modifier tuned each terminator in a different way. However, some modifiers were found to have a similar tuning effect across many different core terminators (Fig. 3c). Some had a generally positive influence (e.g., M21) or negative influence (e.g., M20). Furthermore, the U- and A-tract interactors generally exerted opposite tuning effects on stronger terminators and weaker terminators, tuning them up and down in strength, respectively. Therefore, when tuning a T7 RNAP terminator, while bespoke modifiers are likely required, our library offers some starting points for features that are likely to have a desired effect.

### Insulating transcriptional valves from local genetic context

It has been shown for many types of genetic part that more reliable performance can be achieved by inserting random non-coding sequencers around a part to insulate its function from potential interactions with other nearby sequences^8,17,36^. Our library specifically included random non-coding modifiers of varying length to assess the insulating effects for transcriptional valves. In general, we found that an increase in the length of these modifiers led to a reduction in *T*_e_ variability when an identical valve design was used across numerous genetic contexts (i.e., upstream spacer sequences; Fig. 3d). This matches findings for bacterial promoters and terminators where longer upstream insulating sequences resulted in more predictable gene expression^17,36^. Notably, these effects were also terminator-specific, with some core terminators showing more predictable behavior across modifiers (e.g., T16) than others (e.g., T10) (Fig. 3f). This suggests that some terminators are better suited to tuning T7 RNAP in vitro, while others are better placed to maintain a consistent termination efficiency.

### Exploring modifier-terminator base-pairing

**Fig. 4:**
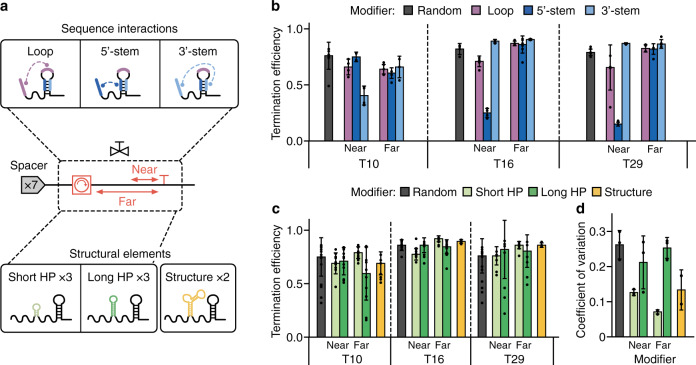
Engineering modifiers that tune and insulate core terminators. **a** Overview of the modifier library based on sequence interactions and structural elements used to explore tuning and insulation of core terminator function. A ‘near’ and ‘far’ modifier variant was designed for each motif (except for the random and structure options). Modifiers were designed to interact with the core terminator via sequence (upper) or structure (lower). For sequence interactions, 8 nt sequences were designed to be complementary to regions of the core terminator covering the loop (purple), 5′-stem (dark blue), 3′-stem (light blue). For structural elements, 3 short hairpins (light green), 3 long hairpins (dark green) and 2 further RNA structures (orange) were designed. **b** Termination efficiency (*T*_e_) for each valve designed to interact via sequence, grouped by modifier. Bars denote median *T*_e_ values ± standard deviation (SD) and from left–right *n* = 6, 4, 2, 4, 5, 4, 3, 5, 4, 4, 3, 3, 6, 2, 7, 4, 3, 3, 4, 4 and 4. **c** *T*_e_ for each designed structural element. Bars denote median *T*_e_ values ± SD and from left–right *n* = 9, 10, 13, 12, 10, 17, 6, 14, 11, 9, 12, 14, 3, 8, 11, 8, 7 and 17. **d** Coefficient of variation of *T*_e_ values for the structural elements. CV is calculated across spacers for each terminator and grouped by structural element. Bars denote mean CV values ± SD and from left–right *n* = 3, 3, 3, 3, 3 and 2. Source data are provided as a Source Data file.

It was evident from this initial library that the general modifiers we had used limited our ability to understand the role of key interactions between the modifier and terminator parts because no terminator specific interactions had been designed. To rectify this, a further library was built to understand the effect of the modifier region upstream of the core-terminator in a more comprehensive way (Fig. 4a). Informed by our findings that longer modifiers were better insulators of *T*_e_ (Fig. 3d), we designed all-new modifiers as length 45 nt to enhance the robustness of the valves function across different genetic contexts. Co-transcriptional folding simulations had highlighted that the sequences we had designed to interact with the U-tract and A-tract were insufficient. These modifiers seldom formed structures that would influence termination by virtue of base-pairing because the A-tract is often short (<6 consecutive A’s) and at the point of termination, the U-tract is sequestered by the RNAP. Therefore, we designed modifier sequences that would target specific sequences within three strong core terminators (T29, T16, T10).

Motifs containing an 8 nt reverse complement sequence of three different regions of the core terminators were designed into modifiers. Gaps were filled with non-structural RNA sequences (see “Methods” section). While ideally we would have used identical padding sequences, we instead chose padding sequences with identical RNA secondary structure (i.e., no predicted structure) as these unique sequences would help ensure accurate read demultiplexing after nanopore sequencing. These motifs targeted the 5′-stem, loop and 3′-stem regions of the terminator hairpin (Fig. 4a). Two variants of each motif were designed to explore the distance dependence of the engineered motifs: “near” which was incorporated into the modifier at its 3′-end (~30 nt from the U-tract), and “far” which was incorporated at the 5′-end of the modifier (~70 nt from the U-tract).

Characterization of this library revealed that base-pairing can significantly reduce termination efficiency in a distance-dependent manner (Fig. 4b). The motifs designed to base-pair with the loop consistently caused a reduction (13–16%) in *T*_e_. Loop-modifier interaction during termination could alter the core terminator hairpin during its formation, at the point of termination, or both, affecting the probability of a termination event. The largest effect on *T*_e_ was caused by motifs designed to interact with the stem of the terminator. We hypothesize that this is because core terminator stem base-pairing is essential for hairpin formation whereas the loop can base-pair with an interacting motif at the same time as the completing hairpin. Therefore, a motif that can base-pair with the stem could outcompete the core terminator hairpin.

For the two strongest terminators (T29, T16) the 5′-stem interactor caused a large drop in *T*_e_, while the 3′-stem interactor did not. In the case of T16 this is likely because 5 of the 8 targeted nucleotides are predicted to be concealed within the T7 RNAP (2 nt for T29 and 3 nt for T10), where they cannot base-pair at the point of termination since they are in the U-tract. This effect on *T*_e_ was greater than any other drop caused by a modifier tested so far. As with previous modifiers, T10 behaved differently to T16 and T29. The 3′-stem interactor had a large effect on termination, while the 5′-stem interactor did not. This could be a consequence of the extra native sequence context between the hairpin and the motifs (5 nt and 3 nt more than T29 and T16, respectively) resulting in a location in which the motif can base-pair with the 3′-stem. However, it may also arise from the inherent tunability of T10. In either case, the effect on *T*_e_ of motifs that base-pair with terminators diminishes when they are placed far from the terminator.

### Exploring structural interactions

It has been shown that structure can stabilize the activity of genetic parts^1,57^. Therefore, we looked to further investigate whether RNA structure could insulate terminator function by designing and testing a library of modifiers containing secondary structure motifs (Fig. 4a). To explore the effect of upstream structure on termination, we designed three short (stem length 3 nt) and three long (stem length 6 nt) hairpins. The sets of short and long hairpins contained one of three loops (UUCG, GAAA, GAGA) known to facilitate strong hairpin formation^58^. Furthermore, modifiers were designed with all of these secondary structures at both the 5′-end and 3′-end to test the distance-dependence of secondary structure influence on termination efficiency. Again, gaps were filled with non-structural RNA sequences (see “Methods” section). A variety of other more complex RNA secondary structures are known and one modifier containing an “elbow” (the TAR element) and one containing a pseudoknot were also designed to see any role these might play^59^.

After assembling and characterizing this new library, we were able to confirm that RNA secondary structure upstream of terminators affected the robustness of terminator function with T7 RNAP (Fig. 4c), similar to that reported previously for *E. coli* RNAP^60^. We found that short hairpin structures and complex RNA structures were the best insulators of terminator function (Fig. 4d), while long hairpin structures made termination efficiency more sensitive to upstream genetic context. The rigid requirements of short hairpin formation mean that they are likely rarely influenced by base-pairing with upstream structure. This would explain why they are good insulators since they offer dependable upstream secondary structure that does not base-pair or interact structurally with the terminator hairpin. In contrast, since long hairpins can form with a variety of stem lengths they could influence and be influenced by neighboring sequences. The resultant diversity of secondary structures that can then arise upstream of the terminator hairpin would mean that these modifiers significantly affect *T*_e_ and therefore act as poor insulators, as seen in our results.

Since we only tested stronger terminators, conclusions cannot be drawn on the capacity of base-pairing and structural sequence motifs to increase *T*_e_. Nonetheless, these results revealed motifs that could alter the *T*_e_ or robustness of terminator function and therefore should be considered when designing genetic circuits that involve uncharacterized gene-terminator combinations.

### Understanding core terminator design principles

**Fig. 5:**
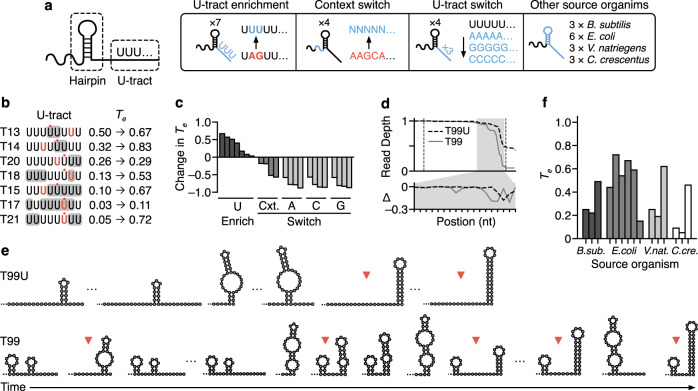
Exploring design features of the core terminator. **a** Overview of modifications made to core terminators, tested without spacers or modifiers: (left to right) U-tract enrichment to include up to 8 consecutive U residues down-stream of the core terminator hairpin; Context switch with the 19–25 nt immediately upstream of the terminator hairpin changed to a random sequence; U-tract switch to consecutive A, C, or G residues; Other source organisms used to find diverse terminator sequences. **b** Effect of U-tract enrichment on most common termination position and change in termination efficiency (*T*_e_). Substitution of nucleotides for U are indicated with gray shading. Termination position before the substitution is indicated in bold red text. Termination position after substitution is indicated with red dot. **c** Change in *T*_e_ for U-tract enrichment (U Enrich, dark gray), context switch (Cxt, gray) and U-tract switches (A, C, G, light gray). **d** Normalized dRNA-seq read depth profiles for the T7 phage T-theta terminator (T99, gray solid line) and a variant (T99U, black dashed line). Dotted lines denote the start and end of the core terminator. The gray-shaded region is expanded in the lower panel showing the change in normalized read depth at each nucleotide position with respect to the previous nucleotide (Δ). **e** Secondary structures predicted by co-transcriptional folding simulation of terminator formation (left to right) for T99U and T99. Positions where measured termination occurs are indicated with red triangles. **f** Measured *T*_e_ of T7 RNAP for terminators sourced from diverse organisms. Source data are provided as a Source Data file.

Our results had shown that many of the relationships observed were terminator dependent, and so a final library was designed and tested to investigate variations of the core terminator part that had the greatest influence on *T*_e_ (Fig. 5a). We constructed a library of designs including U-tract variants, native context variants, and terminators from diverse organisms. This set of core-terminators was assembled and tested in just one upstream genetic context (the *gfp* gene) and so our analysis is focused on understanding the influence of sequence context proximal to the core-terminator hairpin.

Analysis showed that the *T*_e_ of weak core-terminators used in our initial library could be increased by increasing the number of U’s in the U-tract (Fig. 5b). Our initial results indicated that a U-tract of at least 5 U’s consistently resulted in termination (Fig. 2c). Therefore, we re-engineered weaker core-terminators so that they contained a U-tract of length 8 nt. This increased *T*_e_ to varying extents (Fig. 5b). Despite each of these newly designed terminators having the possibility to terminate at a U-tract complete with 8 U’s, the dominant transcript isoform had only 4 or 5 U’s in each case. Nonetheless, the increase in *T*_e_ showed some correlation with the number of extra U’s in the dominant U-tract (Supplementary Fig. 9). For active terminators, the largest increases in termination occurred when additional U’s increased the number of U’s in the dominant transcript isoform to 4 or 5 (T14, T15, T18). Finally, in two cases (T17 and T21), termination of inactive terminators was found to be rescued.

The location of termination and thus the specific transcript produced consistently changed following these modifications (Fig. 5b). For these designs, transcript isoforms became either 1–2 nt shorter and longer. The location of termination and termination prior to a complete “UUUUUUUU” U-tract may arise since the extra U’s were not added to the dominant transcript variant. Instead, they were added at positions that would give a sequence of 8 U’s with the minimal number of single nucleotide substitutions. Therefore, to generate strong terminators from a template sequence, our results suggest first characterizing the dominant transcript form and then increasing the number of U’s within its U-tract.

To complement the modifier library, the same core terminators (T10, T16, T29) were used as a basis for various U-tracts containing no U’s. Variants of each of these core terminators with an 8 nt tract of A, C, or G were designed. To ensure their transcripts could be distinguished after dRNA-seq we put unique non-structural RNA sequences as barcodes upstream of the core terminators (see “Methods” section). These variants were inspired by data showing that T7 RNAP can slip and terminate at sites of 8 consecutive A’s in vitro^59,61^. However, we found this not to be the case for T29, T16, T10, or the T7 phage T-theta terminator (Fig. 5c). The poly-C tract showed very weak termination (*T*_e_ < 0.1). For our previous designs, some native sequence context immediately upstream of the terminator hairpin (and before the modifier) is retained. Changing this decreased *T*_e_ for all the terminators studied (Fig. 5c), indicating that to maintain *T*_e_, there is an optimal position upstream of core terminators to add modifiers.

Characterization of the T7 phage terminator (T-theta) revealed wide diversity in the points of termination (Fig. 5d). We found T-theta to be strong (median *T*_e_ = 0.82) and tunable. The progression of minimum free energy structures predicted to form as each nucleotide is transcribed suggests that a variety of structures form approaching the point of maximum termination (Fig. 5e). This is likely to account for the ability for termination to occur at various positions. Changing the native context immediately upstream of the core-terminator hairpin decreased *T*_e_ by 59%. This was despite a change to the “GC” at the end of the U-tract to “UU”. Furthermore, these modifications to the terminator changed the distribution of transcript isoforms significantly, resulting in a single peak of termination (Fig. 5d). This native context variant changes the co-transcriptional structures predicted in the buildup to the point of maximal termination, preventing a hairpin immediately upstream of the U-tract from forming for the first two possible transcript variants (Fig. 5e). These insights into how upstream genetic context influences terminator hairpin structures are potentially important for not only tuning *T*_e_, but also transcript isoform abundances.

T-theta was also tested with a variety of upstream modifiers designed to base-pair with the core-terminator or form secondary structures. Of these, one short hairpin near to the core-terminator (M81) tuned the ratios of transcript variants depending on the upstream genetic context. Co-transcriptional simulations of this valve indicated that a variety of secondary structures can form immediately upstream of the terminating hairpin, which can extend and therefore be influenced by sequences further upstream. The effect of versatile secondary structures upstream of the valve could potentially influence the transcripts produced as well as *T*_e_. The ability for T-theta to form a complex mixture of transcript variants whose ratio can be tuned by upstream sequence could arise from co-evolution of this terminator with the T7 RNA polymerase. This would result in a high capacity for tuning both transcription (via *T*_e_) and mRNA stability (via RNA degradation) following mutation of the core-terminator or upstream sequence.

Finally, strong terminators highlighted by previous studies were also characterized (Fig. 5f). These comprised a set of three strong (in their host) core-terminators from four different bacteria characterized by Lalanne et al.^4^, along with 3 further *E. coli* terminators with long U-tracts^7^. At least one example of a terminator with *T*_e_ > 0.5 in vitro was present in the selection for each organism. This selection sought to expand the options for engineering strong T7 RNAP valves. While each of these terminators has evolved to function in different cellular contexts, we found that they behave similarly with T7 RNAP in vitro: termination invariably occurred in a region with multiple U’s in the U-tract downstream of a hairpin. These results highlight that terminators sourced from many organisms can terminate T7 RNAP and provide yet more options for core terminator parts when designing valves.

### Controlling expression stoichiometry of a CRISPR guide RNA array

The ability for our valves to control the stoichiometry of transcript isoforms makes them ideally suited for multiplexed regulation of RNA-based parts. To demonstrate how this might be achieved, we chose to focus on the expression of a CRISPR–Cas9 guide RNA (gRNA) array. While gRNAs have been co-expressed as arrays^62–66^, few efforts have been made to rationally regulate the relative levels of gRNAs within an array. This could be important for implementing complex patterns of gene activation or repression. Promoters of varying strength have been used to achieve a similar goal^67^. However, promoters do not couple gRNA stoichiometries to one another in the same way as can be achieved by using transcriptional valves and are sensitive to noise and genetic context that can affect each promoter independently.

**Fig. 6:**
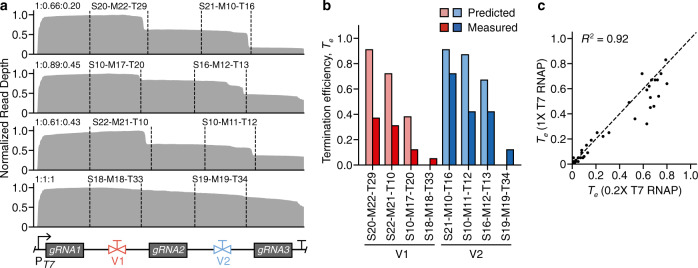
Using transcriptional valves to regulate an array of CRISPR sgRNAs. **a** Normalized dRNA-seq read depth profile for each array. Dotted lines denote the start and end of each valve. General array design is shown at the bottom, with the P_*T7*_ promoter followed by 3 guide RNAs (gRNAs) separated by two transcriptional valves (V1, V2). Resulting gRNA stoichiometries are indicated in top left of each profile (gRNA1:gRNA2:gRNA3) and valve designs are shown above their respective parts. **b** Comparison of predicted (lighter shading) and measured (darker shading) termination efficiency (*T*_e_) for each valve in the arrays. Bars are colored by valve position. **c** Comparison of *T*_e_ measurements for in vitro transcription reactions performed with varying concentrations of T7 RNA polymerase (RNAP). Each point corresponds to a transcriptional valve. *R*^2^ is the square of the Pearson correlation coefficient. Source data are provided as a Source Data file.

We designed four arrays, each containing the same three gRNAs (complete with handles) separated by two unique valves (Fig. 6a; see “Methods” section). A set of designs were selected from the initial valve library (Fig. 3) to give a range of gRNA expression stoichiometries. We pooled the arrays and used T7 RNAP to transcribe the pool in vitro and then performed dRNA-seq characterization to calculate the ratios of expressed gRNAs from each array. We found that as expected each design produced different stoichiometries of the gRNAs (Fig. 6a). We calculated predicted ratios based on the characterization of the valve library and compared those to the measured ratios from the arrays (Fig. 6b). While valves ranked the same in terms of *T*_e_, the absolute termination observed was significantly lower in the array, with a decrease that correlated with proximity to the promoter. This feature has been previously observed^68^, though to our knowledge the cause is not fully understood. One hypothesis is that proximity to the promoter has been predicted to increase transcriptional read-through of protein “roadblocks” by virtue of an increased force from RNAP traffic, which is cumulative^69^, and a similar effect could be occurring in our case.

To test this hypothesis further, we characterized a small library of 40 terminators using varying concentrations of T7 RNAP for the in vitro transcription reactions to vary the RNAP traffic present on the DNA (1× and 0.2× concentrations). We found a strong correlation (*R*^2^ = 0.93) in the *T*_e_ values. However, for some terminators *T*_e_ significantly decreased at the higher concentration of T7 RNAP (Fig. 6c). Therefore, the systematic reduction in *T*_e_ for the CRISPR gRNA arrays, may be a result of the much closer position of the promoter to the terminator in these constructs or other affects our normal termination assay does not capture. Nevertheless, these results demonstrate the ability to use transcriptional valves as a means of multiplexed regulation of RNA-based parts.

## Discussion

In this work, we have shown how transcriptional terminators can be repurposed as “valves” to regulate the flow of RNAP along DNA and control the ratio of transcript isoforms produced. By developing a nanopore-based dRNA-seq characterization approach (Fig. 1), we were able to simultaneously measure the termination efficiency of an entire mixed pool of 1183 unique transcriptional valves as well as provide nucleotide resolution insights into precisely where termination occurred for each (Fig. 2). We found that all terminators produced multiple transcript isoforms, whose ratio could be tuned with upstream sequences. Such detail is lost with more typical fluorescence-based assays^7,16^, but is essential for developing the low-level biophysical models of genetic parts needed for predictive biodesign workflows^70–73^.

While rich, high-content characterization data can normally only be produced for a small set of samples^9,18,40,74^, the approach presented here circumvents this common limitation and allows us to more systematically explore the genetic design space of a large pooled library and extract several design principles. We show how local sequence context (i.e., modifier sub-sequences) can be used to tune termination efficiency, while the inclusion of sufficiently long insulating sequences (45 nt) at the 5′-end of a core terminator reduces changes in *T*_e_ when the same valve is used in conjunction with different upstream coding sequences (Fig. 3). Furthermore, the successful use of terminators from divergent bacteria to control the viral T7 RNAP suggests that a similar characterization approach could be used to rapidly develop libraries of transcriptional valves for RNAPs from other organisms^75–77^.

Iterative design-build-test-learn cycles using rapid, combinatorial DNA assembly and in vitro dRNA-seq enabled us to construct two targeted libraries covering a further 600 designs to investigate properties of sequences near to the terminator that influence *T*_e_ (Figs. 4, 5). The sequence upstream of the terminator often evolves to tune *T*_e_, which is strongly influenced when this sequence interferes with terminator hairpin formation. Short hairpins within the modifier sequence can insulate terminators by stabilizing upstream RNA structure though this ability diminishes with hairpin size. Downstream of the terminator, in the U-tract, the sequence determines whether RNAP pauses are sufficiently long to allow for hairpin formation and transcript dissociation^7^. Here, an abundance of U residues was found to be essential, and their composition determined the precise point(s) of termination. Our newly designed valves behaved similarly at differing T7 RNAP concentrations (simulating varying transcription initiation rates) and could regulate ratios of CRISPR gRNAs by expressing them in an array interspersed with valves (Fig. 6). Our results suggest that increased T7 RNAP traffic, whether caused by absolute T7 RNAP concentration or proximity to the promoter, may cause terminator read through and a decrease in termination in vitro. Therefore, for predictive use of valves, T7 RNAP traffic should be taken into consideration and could offer a mechanism for dynamic control of circuit behaviors in response to cellular processes engineered to regulate T7 RNAP concentration or upstream sequence length^78^.

Although dRNA-seq opens new avenues for high-throughput characterization of genetic part libraries, some challenges remain. The most prominent of these is ensuring the read depth profiles accurately represent the transcript variants present. Here, we show how some unwanted features caused during the preparation of a sequencing library can be effectively corrected for using a simple mathematical model (Supplementary Note 3), but improvements in the ability to sequence full length transcripts would lead to more representative read profiles and would be a valuable direction for future research and will be essential for the comprehensive exploration of large genetic design landscapes^79^.

The focus of this work was to assess the function of transcriptional valves in vitro. This allowed us to avoid confounding factors that would be difficult to control for in vivo (e.g., RNA degradation^20^). However, a detailed assessment of how well these results hold or correlate to in vivo measurements would offer another interesting future direction and such a study could also be carried out in high-throughput using other sequencing-based approaches that combine fluorescence-activated cell sorting (FACS) and subsequent sequencing (Sort-seq)^70,80^ or targeted approaches based on the pull-down of specific RNAs.

This work views transcriptional terminators in a new light. Not merely as a hard endpoint when producing a transcript, but as a means to tune and orchestrate one of the many flows (e.g., transcription and translation) that underpin the synthesis of proteins from DNA. Nature is known to regulate gene expression at multiple levels and through numerous processes to create complex regulatory programs^81^. Transcriptional valves offer bioengineers a perspective on how multi-gene regulation can be implemented at a purely transcriptional level and a means to implement more diverse information flows in genetic circuitry.

## Methods

### Strains and media

All cloning was performed using *Escherichia coli* strain DH10-β (F^–^ *endA*1 *glnV*44 thi-1 *recA*1 *relA*1 *gyrA*96 *deoR nupG purB*20 φ80d*lacZ*ΔM15 Δ(*lacZYA*–*argF*)U169, *hsdR*17(r_K_^–^m_K_^+^), λ^–^_­_) (C3019I, New England Biolabs). Cells were grown in LB Miller broth (L3522, Sigma-Aldrich). Antibiotic selection was performed using 100 µg/mL of ampicillin (A9393, Sigma-Aldrich).

### Pooled combinatorial assembly of a transcriptional valve library

The pGR plasmid backbone^7^ was modified for use in combinatorial assembly by first mutating the *gfp* stop codons from “TAATAA” to “TTAGCA” using Q5 mutagenesis (E0554S, New England Biolabs) and second replacing the *araC* gene and P_*BAD*_ promoter with a consensus T7 promoter sequence (pT7, Supplementary Table 1). The pGR plasmid was a gift from Christopher Voigt (Addgene plasmid #46002). Promoter substitution used the restriction enzymes AatII (10 units; R0117S, New England Biolabs) and NheI-HF (10 units; R3131S, New England Biolabs) in 1× CutSmart buffer (B7204S, New England Biolabs) and nuclease-free water (final volume 50 µL) at 37 °C for 30 min; 80 °C for 20 min, followed by adding annealed inserts (Supplementary Table 1) to vector DNA (0.020 pmol) at a 3:1 molar ratio with T4 DNA ligase (400 units; M0202S, New England Biolabs), T4 DNA ligase buffer and nuclease-free water to a final volume of 20 µL for 30 min at room temperature and then 65 °C for 10 min.

Oligonucleotides (25 nmol, dry lyophilized solid, standard desalting; Integrated DNA Technologies) were diluted to 100 µM in TE buffer (10 mM tris(hydroxymethyl)aminomethane, 0.1 mM ethylenediaminetetraacetic acid, pH 8.0). Duplex DNA was assembled for each core terminator, modifier, or spacer variant by annealing the complementary forward and reverse oligonucleotides (2 µL, 100 µM) in 46 µL annealing buffer (10 mM Tris, pH 7.5–8.0, 50 mM NaCl, 1 mM EDTA), heating to 95 °C for 5 min and slowly cooling to room temperature. Duplex DNA of all variants (1 µL (1 pmol) of each) was combined, and the pool was diluted to a final concentration of 1 pmol/µL with nuclease-free water. The pooled duplex DNA (20 µL) was phosphorylated using T4 polynucleotide kinase (10 units; M0201S, New England Biolabs) in 10× T4 DNA ligase buffer (2 µL) at 37 °C for 30 min; 65 °C for 20 min. Meanwhile, plasmid backbone DNA (1 µg) was digested using EcoRI-HF (20 units; R3101S, New England Biolabs) and SpeI-HF (20 units; R3133S, New England Biolabs) in 10× CutSmart buffer (5 µL) and nuclease-free water (35 µL) at 37 °C for 4 hr and then 80 °C for 20 min, before gel extraction (0.8 % agarose gel, gel green dye, 80 V, 100 min; T1020S, New England Biolabs Monarch). Plasmid backbone (50 fmol) was used for pooled ligation based combinatorial assembly by combining with a 5-fold excess of the aforementioned phosphorylated duplex DNA pool (containing 250 fmol of each design for insertion into the plasmid), nuclease-free water (40 µL), 10× T4 DNA ligase buffer (5 µL) and T4 DNA ligase (320 units; M0202S, New England Biolabs) and incubating at room temperature for 3 hr and then 65 °C for 10 min.

The pooled transcriptional valve library (3 µL) was then added to each of 12 aliquots of *E. coli* strain DH10-β cells (45 µL each; C3019I, New England Biolabs) thawed on ice for 10 min and mixed gently by tapping. The mixture was left on ice for 30 min and heat-shocked at 42 °C for 30 seconds before leaving for 5 min on ice. NEB 10-beta/Stable Outgrowth Medium (450 µL; B9035S, New England Biolabs) was added to each aliquot and the mixture was shaken vigorously (1250 rpm) at 37 °C for 60 min. Each aliquot was then added to one 1.5 L rectangular glass dish (Pyrex) containing LB agar with ampicillin and cells were grown overnight. Following this, sufficient colonies were scraped from agar plates for >400-fold library coverage and DNA was extracted (T1010L, New England Biolabs). DNA concentrations were measured using a NanoPhotometer N60 (Implen). All libraries constructed in this work are described in Supplementary Table 2.

### Verification of assembled transcriptional valve library

DNA from the pooled transcriptional valve library (400 ng) was prepared for DNA sequencing using the rapid barcoding kit following the standard protocol (SQK-RBK004, Oxford Nanopore Technologies). DNA samples were sequenced for 48 h on FLO-MIN106 flow cells. Generated FAST5 files were basecalled using guppy version 3.1.5. BLASTN version 2.2.31 (with the same parameters as for dRNA-seq demultiplexing) was used to align sequencing reads to reference sequences. DNA sequencing reads were demultiplexed by selecting the best alignment to a design based upon maximum bitscore for each sequencing read. Sequencing reads with no alignment to a design, or alignments to multiple designs with the same maximum bitscore were excluded from further analysis. Part and design frequencies were calculated relative to the total number of annotated sequencing reads.

We used demultiplexed sequencing reads to generate a consensus sequence for each design and assess DNA assembly fidelity. Demultiplexed sequencing reads for each design were aligned to the plasmid encoding the design using minimap2 version 2.17^46^. Racon version 1.4^82^ with parameters: -m 8 -x -6 -g -8 -w 500, was used to polish the plasmid sequence and refine the consensus sequence produced. The polished and reference sequences were then aligned using Multiple Alignment using Fast Fourier Transform (MAFFT) version 7 with parameters: --localpair --maxiterate 1000. Finally, a script was used to count the average number of single nucleotide polymorphisms per design. Annotated plasmid sequences are available in Supplementary Data 1.

### Pooled in vitro transcription and direct RNA sequencing

DNA from the pooled transcriptional valve library (1 ug) was linearized using AatII (10 units) in 10× CutSmart buffer (5 µL) and nuclease-free water (40 µL) at 37 °C for 30 min and then 80 °C for 20 min. Duplicate reactions were purified (T1030L, New England Biolabs) and eluted in nuclease-free water (12 µL). In vitro transcription was performed using HiScribe™ T7 High Yield RNA Synthesis Kit (E2040S, New England Biolabs). The following reagents were combined: T7 RNA Polymerase mix (2 µL), adenosine triphosphate (2 µL), guanosine triphosphate (2 µL), cytidine triphosphate (2 µL), uridine triphosphate (2 µL), kit reaction buffer (2 µL), the RNA calibration strand (0.5 µL) and the linearized DNA pool (250 ng) at 37 °C for 35 min. Synthesized RNA was diluted 20-fold in nuclease-free water and purified (R1013, Zymo Research). The purified RNA (10 µg) was then poly-adenylated using *E. coli* Poly(A) Polymerase (10 units; M0276S, New England Biolabs) with 10× reaction buffer (2 µL), RNase inhibitor murine (0.5 µL; M0314S, New England Biolabs) and 2 µL adenosine triphosphate, ATP (10 mM) at 37 °C for 30 min. The reaction was stopped by proceeding to RNA purification with elution in 15 µL (R1013, Zymo Research). Polyadenylated RNA (1 µg) was prepared using the direct RNA sequencing kit following the standard protocol (SQK-RNA002, Oxford Nanopore Technologies) with inclusion of the reverse-transcription step and flow cell priming kit EXP-FLP002 (Oxford Nanopore Technologies). RNA samples were sequenced using MinKNOW version 20.06.5 for 48 hr on FLO-MIN106 flow cells.

### Computational demultiplexing and analysis pipeline

First, dRNA-seq data in FAST5 format was basecalled using guppy version 3.1.5 in high-accuracy mode. Next, the design sequences (not including plasmid backbone) were aligned to all basecalled sequencing reads using BLASTN version 2.2.31^83^. BLASTN parameters were selected based upon simulated nanopore RNA-seq data: -outfmt 6 -gapopen 5 -gapextend 2 -reward 2 -penalty -3 -evalue 1 -word_size 4 -max_target_seqs 1000000 -max_hsps 1. Python and Bash scripts were then used to match each sequencing read to a design based upon the best alignment (the alignment with maximum bitscore). Sequencing reads with no alignment to a design, or alignments to multiple designs with the same maximum bitscore were excluded from further analysis. Part and design frequencies were calculated relative to the total number of annotated sequencing reads.

Parsed reads were then mapped to a plasmid sequence encoding the appropriate design using minimap2 version 2.17^46^ to generate a sequence alignment (SAM) file. Using pySAM^84^, the SAM file was refined by removing sequencing reads that did not contain a full design sequence and terminated between the start of the spacer and 20 nt into the core terminator as defined by a general feature format (GFF) file for each plasmid sequence bearing a design. Reads from two identical sequencing runs were pooled to calculate final *T*_e_ values. Termination efficiencies were calculated based upon the read depth on either side of the valve (see main text for details) which was further corrected by subtracting the predicted *T*_e_ deviation (Supplementary Note 3; Supplementary Fig. 4).

### Generating non-structural RNA sequences

All regions to provide padding in modifiers were designed using RNAInverse version 2.5, which can generate RNA sequences with a specific RNA structure^85^. A query structure that the tool would accept was submitted: “(……………………………..)”. Then the output sequences were trimmed 1 nt at either end and any sequences with a non-zero folding energy, a restriction enzyme recognition site for *Eco*RI, *Spe*I, or *Aat*II, or a site with 4 or more adjacent identical nucleotides were removed. The remaining sequences were used to fill the gaps required for testing base-pairing and structural modifier sequences in the modifier at different distances from the core-terminator.

### Designing arrays of gRNAs regulated by transcriptional valves

Valve designs used in the arrays consisted of particular sets of spacer, modifier, and core-terminator sequences. The guide sequences were selected from the CRISPRlator construct designed by Santos-Moreno et al.^86^. Since the array would result in multiple guides per transcript, the RNA transcripts would have to be processed following transcription to separate them and make them functional. The same strategy used by Santos-Moreno et al. was used: inclusion of Csy4 recognition sites around each gRNA.

Due to limitations on the number of repetitive sequences that can be effectively synthesized, measures were taken to reduce sequence homology. Csy4 recognition sites were shortened to 15 nt, which includes all but the 3′-cytidine of the shortest functional recognition site to be characterized^87^. The 3′-cytidine was omitted as it falls outside of the hairpin and the Csy4 recognition site used by Santos-Moreno et al. omitted it, yet it is recognized by the enzyme. Instead of using the same CRISPR handle for each gRNA we selected a unique handle for each gRNA from the non-repetitive examples in Reis et al.^63^. Three handles with dissimilar sequences and high functionality were used. These handles are compatible with dCas9sp. While gRNAs are often transcribed with a terminator sequence, this was omitted as valves were used to regulate transcription instead.

At the start and end of each array was a short 15–25 nt randomly generated sequence to allow for PCR amplification and serve as a buffer for restriction enzyme cleavage. Finally, around each gRNA-handle and each valve we included unique single-cutter restriction sites to facilitate modification of the arrays. The complete array sequences were ordered as gBlock gene fragments (Integrated DNA Technologies).

### In vitro transcription and dRNA-seq of arrays

DNA arrived freeze-dried in tubes and was solubilized as follows. The tubes were solvated in nuclease-free water (8 µL), briefly vortexed, incubated at 50 °C for 15 min, cleaned up (Monarch DNA cleanup kit, T1030L, New England Biolabs), and eluted in nuclease-free water (10 µL). After measuring the concentration by nanodrop, all four arrays were pooled (62.5 ng of each) along with the RNA calibration strand (0.5 µL) and transcribed using the NEB HiScribe™ T7 In Vitro Transcription Kit protocol and reagents (E2030, New England Biolabs). The mixture was mixed thoroughly by tapping and incubated at 37 °C for 30 min. 10 µL of the reaction product was diluted 5-fold with nuclease-free water and purified using the Zymo Clean & Concentrate 25 kit (RCC-25, Zymo Research), eluting in nuclease-free water (25 µL). Following RNA quantification (Nanodrop), 10 ug of this RNA was diluted to a total volume of 13.5 µL with nuclease-free water and polyadenylated using *E. coli* Poly(A) Polymerase (10 units), with RNase inhibitor murine (0.5 µL), 10× *E. coli* Poly(A) Polymerase Reaction Buffer (2 µL) and ATP (10 mM, 2 µL). The reaction was incubated at 37 °C for 35 min and stopped by purification with the Zymo Clean & Concentrate 25 kit. Following nanodrop quantification, polyadenylated RNA transcripts (1 µg) were prepared for sequencing using the nanopore kit SQK-RNA002 with inclusion of the reverse-transcription step, flow cell priming kit EXP-FLP002 (Oxford Nanopore Technologies) and sequenced in duplicate on FLO-MIN106D (Oxford Nanopore Technologies) flow cells using MinKNOW version 20.06.5 for 48 h.

### Computational tools and genetic design visualization

Computational analyses were executed using Python version 3.5 using the packages NumPy version 1.18.5, SciPy version 1.5.4, Pandas version 1.0.5, and matplotlib version 3.2.2. All genetic diagrams are shown using Synthetic Biology Open Language Visual (SBOL Visual) notation^88^. SBOL Visual diagrams were generated using the DNAplotlib Python package version 1.0^89^ which were then annotated and composed with OmniGraffle version 7.9.2.

### Reporting summary

Further information on research design is available in the Nature Research Reporting Summary linked to this article.

## Supplementary information

Supplementary Information

Description of Additional Supplementary Files

Supplementary Data 1

Supplementary Data 2

Reporting Summary

## Data Availability

The sequencing data generated by this study have been deposited in the OSF database under 10.17605/OSF.IO/DUSPK. Plasmid sequences for all libraries in FASTA and GFF formats, are available in Supplementary Data 1. The termination efficiency of all transcriptional valves is summarized in Supplementary Data 2. Source data are provided with this paper.

## Source data

Source Data
