# Supplementary material for: Massively parallel characterization of engineered transcript isoforms using direct RNA sequencing
Source: Nat Commun. 2022 Jan 21;13:434. doi: 10.1038/s41467-022-28074-5 (PMC8783025; doi:10.1038/s41467-022-28074-5)
Supplement: Supplementary file 1 — Supplementary Information [file 41467_2022_28074_MOESM1_ESM.pdf]

Supplementary Information for:

Massively parallel characterization of engineered transcript isoforms using direct RNA sequencing

Matthew J. Tarnowski and Thomas E. Gorochowski

Supplementary Notes		Page
Supplementary Note 1:	Library coverage calculation	2
Supplementary Note 2:	Transcription profile features	3
Supplementary Note 3:	Modelling direct RNA sequencing	6
**Supplementary Figures**		
Supplementary Figure 1:	Analysis of library assembly	8
Supplementary Figure 2:	Design of library used to optimize demultiplexing	9
Supplementary Figure 3:	Fitting model to direct RNA sequencing data	10
Supplementary Figure 4:	Deviation between observed and actual termination efficiencies	11
Supplementary Figure 5:	Polyadenylation efficiencies	12
Supplementary Figure 6:	Impact of polyadenylation efficiency on direct RNA sequencing read profiles	13
Supplementary Figure 7:	Comparison of termination efficiencies across experimental replicates	14
Supplementary Figure 8:	Analysis of possible predictors of termination efficiency	15
Supplementary Figure 9:	Effect of U-tract changes on termination efficiency	16
**Supplementary Tables**		
Supplementary Table 1:	Oligonucleotide sequences	17
Supplementary Table 2:	Designed libraries	26
**Supplementary References**		27

Supplementary Note 1: Library coverage calculation.

We estimated library coverage using the approach presented by Patrick *et al.*[4] to calculate the expected number of distinct sequences in a library chosen at random from a set of sequence variants. Given a pooled library containing *L* sequences, and a set of *V* equiprobable variants, let v_i be one of the possible variants. Since the variants are equiprobable, the mean number of occurrences of v_i in *L* is

$$\lambda = L\,/\,V. \tag{S1}$$

For $\lambda \ll L$ (i.e., $V \gg 1$), the actual number of occurrences of v_i in *L* is essentially independent of the number of occurrences of any other variant v_j where $j \neq i$, and therefore well-approximated by a Poisson distribution

$$P(x) = \frac{e^{-\lambda}\lambda^{x}}{x!}, \tag{S2}$$

where *P(x)* gives the probability that v_i occurs exactly *x* times in the library. The probability that v_i occurs at least once is given by $1 - P(0) = 1 - e^{-\lambda} = 1 - e^{-L/V}$. Therefore, the number of distinct variants expected in the library is given by

$$C \approx V(1 - e^{-L/V}), \tag{S3}$$

and the fractional completeness of the library is

$$F\ = \frac{C}{V} \approx 1 - e^{-L/V}. \tag{S4}$$

The library size required for fractional completeness *F* is therefore

$$L\ \approx\ -V\ln(1 - F). \tag{S5}$$

In our case, *V* = 1183 variants and we require a fractional completeness of $F > 1 - \frac{1}{1183} = 0.99915$ to ensure with high probability the representation of all variants in the library. This necessitates a library size of at least $L\ \approx\ -V\ln(1 - 0.99915) = 8364$. To achieve this, we performed a transformation protocol that used 10 large trays with approximately 50,000 transformants per tray (**Methods**), resulting in $L \approx 500000$.

Supplementary Note 2: Transcriptional profile features

After characterization of our initial transcriptional valve library, there were several key features that were present within the generated transcriptional profiles. First, we noticed that dRNA-seq reads often had 6 nt of their 5' sequence truncated (**Supplementary Figure 6a**), which could make it difficult to determine precise transcription start sites. As dRNA-seq progresses from the 3' to 5' end of an RNA molecule, this short region likely corresponds to the point where the motor protein that ratchets the RNA molecule through the pore reaches the 5'-end and releases the molecule, causing an increased error rate or removal of the short sequence still contained within the pore.

Second, we found that all dRNA-seq read depth profiles showed drops in read depth when moving from the 3'- to 5'-end (**Supplementary Figure 6**). Such a feature is found in all nanopore dRNA-seq studies to date covering RNA samples from many different organisms [1,2]. It is thought to arise due to fragmentation of full-length RNA molecules (e.g., by shearing caused during pipetting) and/or premature abortion during sequencing resulting in truncated reads. In contrast, only small drops were observed for nanopore DNA sequencing of the constructs (**Supplementary Figure 6a**), possibly due to the greater stability of the molecule [3].

The small proportion of sequencing reads representing RNA fragmented within the barcodes used for mapping leads to a minority of erroneous read mappings. This occurs where the sequencing read matches only part of the barcode and it is impossible to accurately align that read to a particular combinatorial design. We removed sequencing reads arising from these mapping artefacts by selecting only reads with alignment across the spacer, modifier and the first 20 nt of the terminator. The model we outline later corrects for the removal of these reads. While this means that any drops within this region would be missed, studying the profiles generated without omitting these reads did not reveal any noticeable drops in this region. Termination of T7 RNAP requires both a hairpin structure and U-tract and while both of these elements are found in different modifiers, neither of them are found together, making drops caused by termination highly unlikely. Nonetheless, this presents a limitation for studying combinatorial libraries using this method – only transcriptional drops at the end of the barcode can be studied.

To validate the hypothesized causes of RNA fragmentation and explore their possible impact on T_e measurements, we developed a mathematical model (**Supplementary Note 3**) and used data from an RNA Control Strand (RNA CS) that is externally 'spiked-in' to each dRNA-seq experiment for quality control assessments. Because the RNA CS is a single fixed length sequence, we could use it to test how different amounts of fragmentation or sequencing abortion affect the shape of the read depth profile recovered. We found that experimental data could be

well described by a simple model with three probabilistic processes: fragmentation before ligation of sequencing adapters, successful adaptor ligation, and sequencing read truncation (**Supplementary Note 3**; **Supplementary Figure 3**). Sequencing read truncation could be caused by RNA fragmentation (after adapter ligation) and/or early abortion of the sequencing process. We found that the impact of these effects on T_e was small (**Supplementary Figure 4**).

RNA fragmentation meant that many sequencing reads did not contain an intrinsic barcode. To demultiplex sequencing reads using our pipeline, reads are assigned via best alignment to an intrinsic barcode. Therefore, any reads not containing an intrinsic barcode cannot be mapped to a design. Fragmentation causes a significant reduction in the number of reads mapping to an intrinsic barcode (only ~20% of the total reads had an alignment to a barcode). Therefore, improvements in experimental protocols to reduce fragmentation/truncation or the incorporation of methods to enrich barcode containing reads (e.g., using 'read until' technologies [42] or sequence-specific dRNA-seq) could both improve the accuracy of T_e calculations and increase the size of the libraries that can be assessed using a single sequencing run.

While read profiles for RNA CS decrease only towards the 5'-end, profiles for designs decrease in both directions away from the barcode. It is not clear why this is the case since the RNA CS sequence was included in the *in vitro* transcription reaction and therefore was exposed to the same experimental conditions as our designs. It could reflect increased degradation of *in vitro* transcribed RNA, or a gradual drop-off of T7 RNA polymerase during the process of transcription, both of which would not affect the measured termination efficiencies.

A third observation was that in the case of poor polyadenylation, significant drops in read depth were seen outside of the core terminators and predominantly at short poly-A sequences >3 nt in length (**Supplementary Figure 6**). When preparing RNA for dRNA-seq a poly-A tail is required for ligation of sequencing adapters to the 3'-end of the RNA molecules. As *in vitro* transcription of our constructs will not produce transcripts of this form, we used *E. coli* poly(A) polymerase to polyadenylate all the RNAs produced (**Methods**). Analysis of the dRNA-seq data showed <10 nt poly-A tails were present, which were shorter than other dRNA-seq runs we had previously performed (**Supplementary Figure 5**).

We hypothesized that inefficient polyadenylation allows for fragmented RNAs with a short poly-A end to become enriched during sequencing and thus causes notable drops at these points within a construct that do not correspond to termination events. Our subsequent dRNA-seq runs with efficient polyadenylation do not show these drops in read depth at adenosine homopolymer regions. For runs with inefficient polyadenylation (not characterized in this paper), we could partially correct read profiles for designs containing parts with poly-A regions in their template

strand (i.e., I10, T13 and T27) by retaining only mapped reads which do not terminate at a poly-A motif outside the terminator hairpin (**Supplementary Figure 6c**). However, even with this correction, T_e measurements were significantly affected for all designs (**Supplementary Figure 6d**) and therefore we repeated these experiments with efficient polyadenylation.

Supplementary Note 3: Modelling direct RNA sequencing

We developed a simple probabilistic model to capture the key processes impacting the reads recovered from a direct RNA sequencing (dRNA-seq) run. The following figure provides an overview of the major steps.

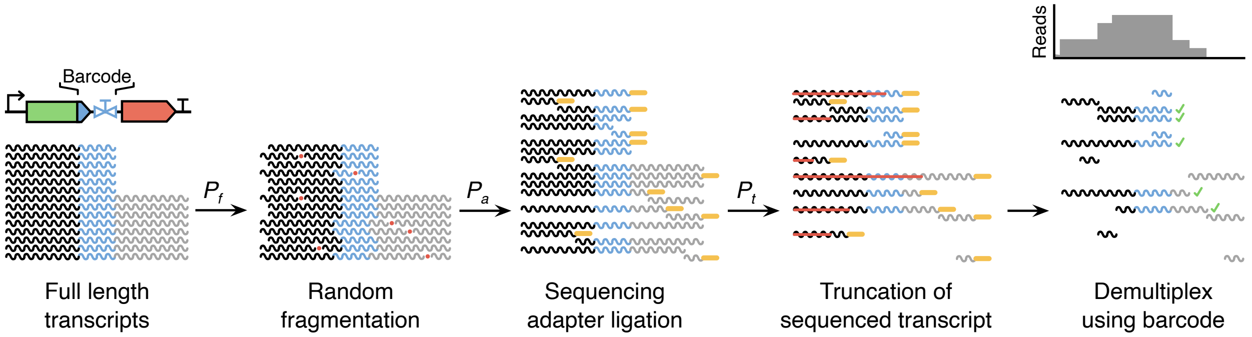

Overview of the direct RNA sequencing model. Reads are denoted by squiggles that are color coded to show core regions (e.g., blue region is the intrinsic barcode). Red dots show points of random fragmentation, orange oblongs represent sequencing adapters attached to only the 3'-end of an RNA molecule, and green ticks denote reads that contain a complete barcode sequenced and which are used to generate a read depth profile. P_f, P_a, and P_t are probabilities that reads are selected for each of the modification steps (i.e., random fragmentation, adapter ligation, and truncation, respectively).

We begin by assuming that all starting RNA transcripts correspond to either an isoform that terminates at the transcriptional valve or at an appropriate point downstream of the valve. First, reads are chosen with probability P_f to become fragmented at a random location along their length. This step captures the inevitable fragmentation that occurs when extracting and purifying an RNA sample. Next, sequencing adapters are attached to full length transcripts and fragmented RNAs with probability P_a and only molecules with an adapter attached are taken forward for sequencing. Sequenced molecules are then chosen with probability P_t for truncation at a random position along the sequence. This step captures possible further fragmentation of the RNA during sequencing library preparation whereby only the fragment containing the adapter is sequenced, or possible truncation of reads due to premature termination during the sequencing of a molecule. In both cases, this significantly reduces the information captured per read and renders many reads impossible to demultiplex when truncation occurs downstream of the intrinsic barcode. Finally, we filter out any that do not contain a complete transcriptional valve design (i.e., intrinsic barcode). Reads without a full barcode cannot be uniquely identified and so the reads are removed during the demultiplexing step. Reads that make it through these steps are then used to generate a read depth profile.

To demonstrate the model's ability to capture read depth profiles generated from real sequencing data, we made use of the RNA Control Strand (CS) that is externally 'spiked-in' to all dRNA-seq runs for Quality Control (QC) purposes. The RNA CS is a single known sequence unlike any other in our library and only consists of full-length RNA molecules. Fitting our model to dRNA-seq data from the two biological replicates, we found that parameter values of P_f = 0.1, P_a = 0.66 to 0.90 (depending upon the sequencing run) and P_t = 0.45 enabled a close fit for all sequencing runs, with only minor deviations at 5' and 3' ends of the RNA CS sequence (**Supplementary Figure 3a**). We also assumed the presence of an intrinsic barcode in the center of the RNA CS sequence and found that our model could also accurately predict read depth profiles recovered after demultiplexing of the real dRNA-seq data (**Supplementary Figure 3b**). This suggests that the read distribution that is generated by the model closely fits that recovered from sequencing.

Finally, to assess how well the observed read depth profiles matched the ground truth, we used the model with parameters fitting to the real dRNA-seq data for RNA CS to simulate the sequencing process on synthetically generated transcripts for a hypothetical set of transcriptional valves with termination efficiencies varying between 0 and 1. By comparing the actual termination efficiency of each hypothetical valve with the observed termination efficiency measured from the generated read depth profiles, we found a slight over estimation in T_e (**Supplementary Figure 4**). To ensure this didn't bias our measurements for the data from the real transcriptional valves, this deviation was corrected for by subtracting the calculated error from the observed termination efficiency seen in the model simulations, to give a final T_e value. Though P_t varied between sequencing runs, the error correction for any given T_e value was found to be consistent across sequencing runs (±1% deviation).

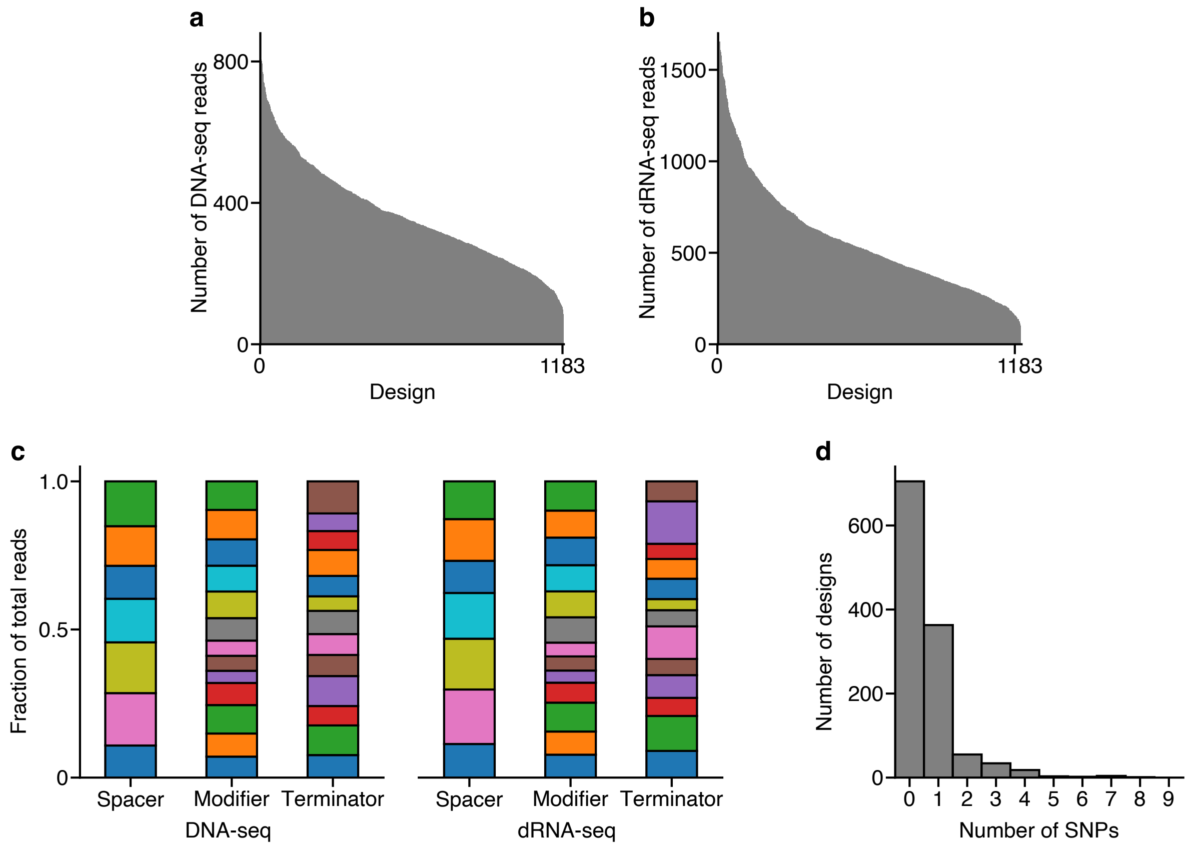

Supplementary Figure 1: Analysis of library assembly. (**a**) Number of DNA-seq reads for each design, ordered by number of reads. (**b**) Number of dRNA-seq reads for each design, ordered by number of reads. (**c**) Frequency of each part in the DNA-seq (left) and dRNA-seq (right) data. Part and design frequencies were calculated relative to the total number of annotated sequencing reads. (**d**) Number of single nucleotide polymorphisms (SNP) per design.

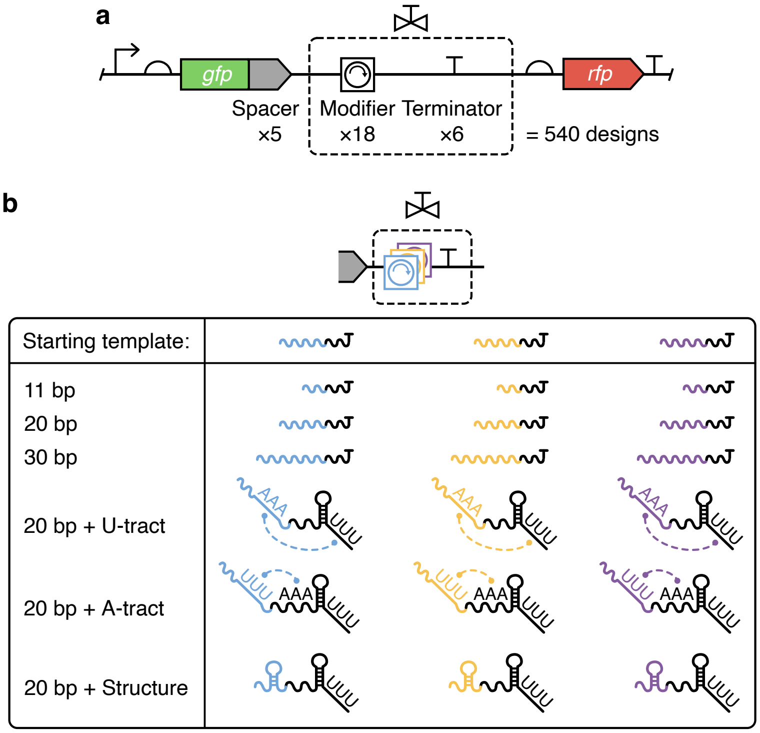

Supplementary Figure 2: Design of library used to optimize demultiplexing. (**a**) The library consists of 5 spacers (S1–S5), 18 modifiers (all parts with references beginning with M1–M3) and 6 terminators (T2–T7), resulting in 540 unique designs. For part sequences see **Supplementary Table 1**. (**b**) Modifiers were based upon 3 random starting template sequences, represented by different colored subsequences. From each template sequence 6 variants were made, each containing different proportions of the template sequence indicated by the number of base pairs: 11 bp sub-sequence, 20 bp sub-sequence, full 30 bp sequence, a 20 bp sub-sequence with U-tract interactor motif, a 20 bp sub-sequence with A-tract interactor motif, a 20 bp sub-sequence with structural motif.

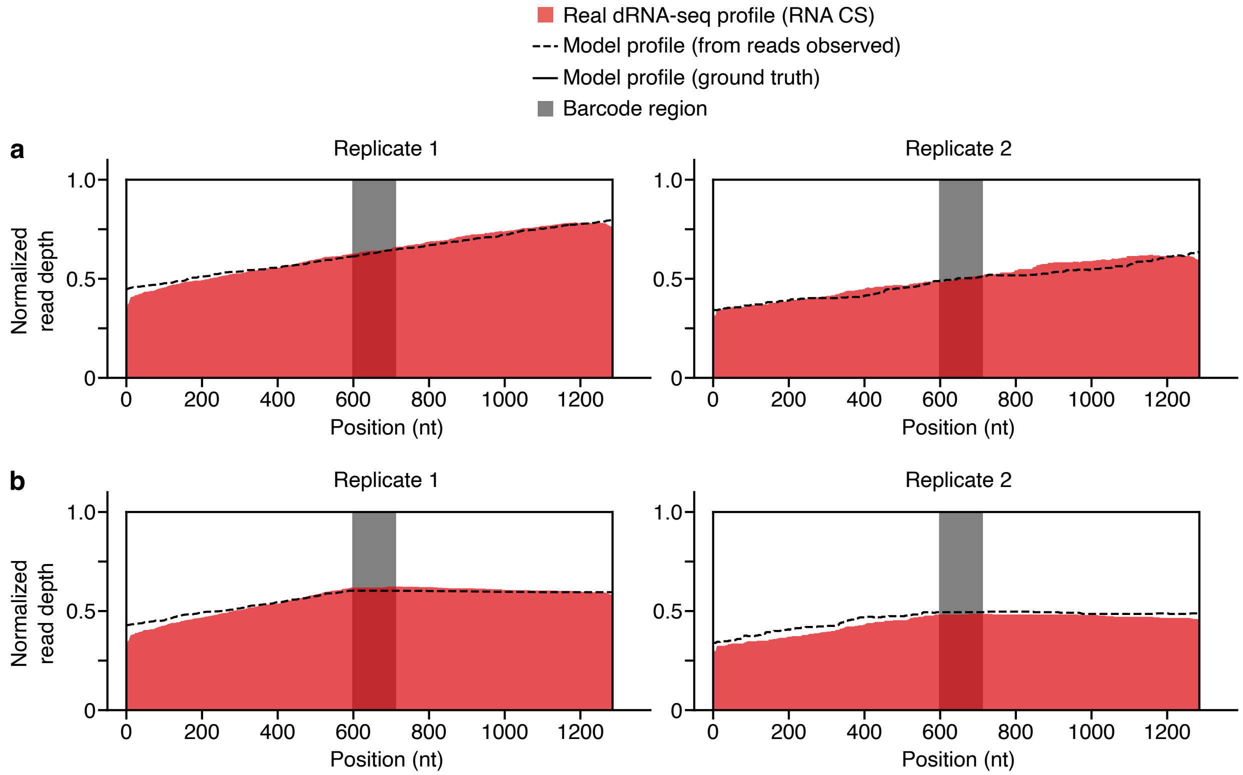

Supplementary Figure 3: Fitting model to direct RNA sequencing data. (**a**) Read depth profiles shown for all reads mapping to the RNA CS sequence for two dRNA-seq biological replicates (filled red) and fitted dRNA-seq model used to simulate the processing of the total number of reads with a BLASTN alignment to the RNA CS sequence, where $P_f = 0.1$, $P_a = 0.8$, $P_t = 0.45$ (dashed black line for observed profile, solid black line for the model ground truth). (**b**) Read depth profiles for reads that map to the grey 'intrinsic barcode' for the real dRNA-seq data (filled red) and fitted model (dashed black line for observed profile, solid black line for the model ground truth). The termination efficiency for the RNA CS 'intrinsic barcode' is zero.

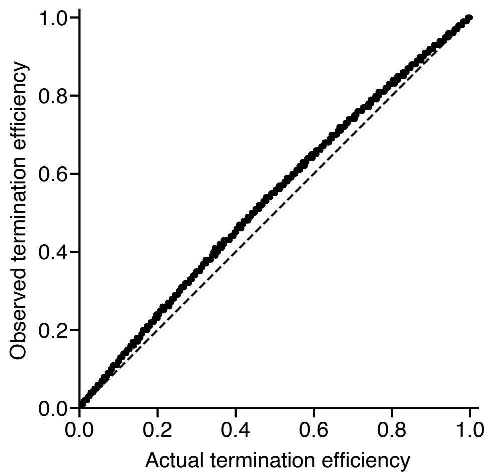

Supplementary Figure 4: Deviation between observed and actual termination efficiencies. Each point denotes a model simulation based on 100,000 simulated reads for transcriptional valves with varying termination efficiencies and parameter values of P_f = 0.1, P_a = 0.87 and P_t = 0.45 (**Supplementary Note 2**). Dashed line shows $y = x$.

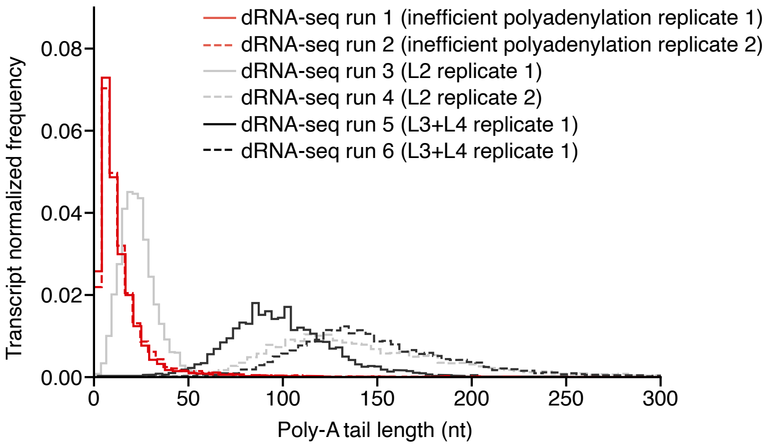

Supplementary Figure 5: Polyadenylation efficiencies. Histograms showing the varying lengths of RNA poly-A tail lengths for several sequencing libraries prepared in this work (**Supplementary Table 2**). RNA from L3 and L4 were pooled and prepared together as a single sequencing library.

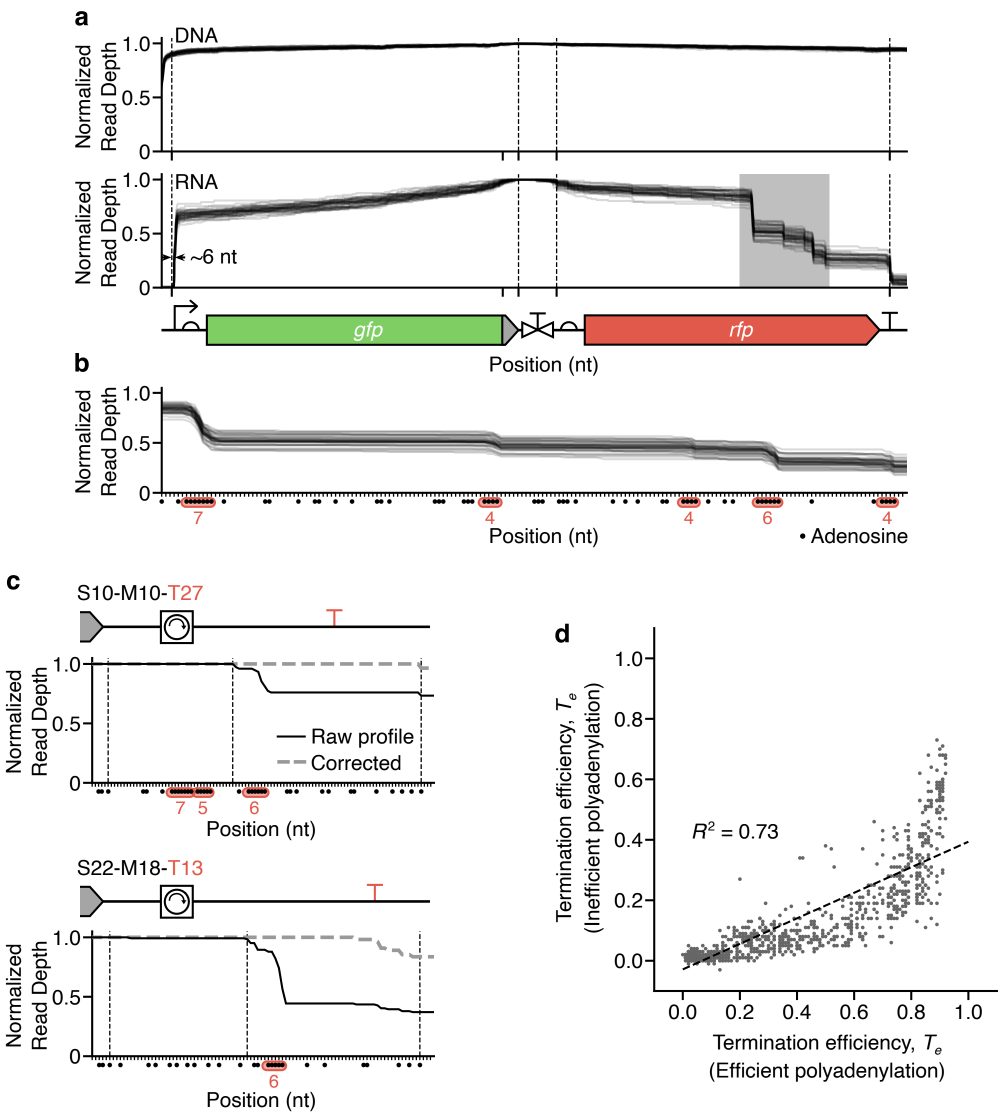

Supplementary Figure 6: Impact of polyadenylation efficiency on direct RNA sequencing read profiles. (**a**) Normalized sequencing read depth profiles from nanopore-based DNA-seq and dRNA-seq for 42 designs containing the same core terminator T33 (non-terminating control) and modifiers of length 30 nucleotides. Vertical dotted lines denote transcript and valve boundaries. Plasmid map illustrated beneath, to scale. Grey shaded region is expanded in the panel below. (**b**) Expanded region from panel A showing dRNA-seq read depth profiles with dots corresponding to adenosine nucleotides. Adenosine homopolymers >3 nt in length are highlighted in red and their lengths are shown below. (**c**) Corrected (dashed grey lines) and raw (solid black lines) dRNA-seq read depth profiles for two different designs where the core terminator contains an adenosine homopolymer within the terminator sequence. Vertical dotted lines indicate spacer-modifier and modifier-terminator boundaries. (**d**) Comparison of estimated termination efficiency of designs from library L2 with and without efficient polyadenylation during sequencing library preparation.

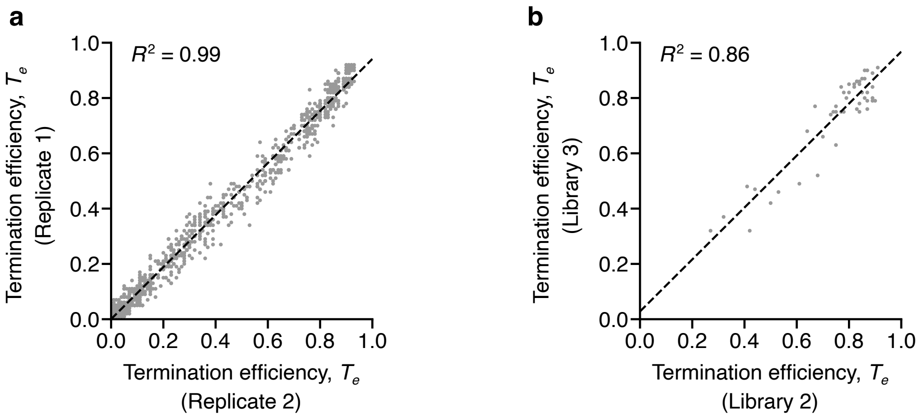

Supplementary Figure 7: Comparison of termination efficiencies across experimental replicates. (**a**) Comparison of termination efficiency between experimental replicates of the same library (L2). (**b**) Comparison of termination efficiency of constructs shared between two different libraries (L2 and L3). Each point represents a single transcriptional valve design and dotted line shows the linear regression. R^2 is the square of the Pearson correlation coefficient. See **Supplementary Table 2** for library compositions.

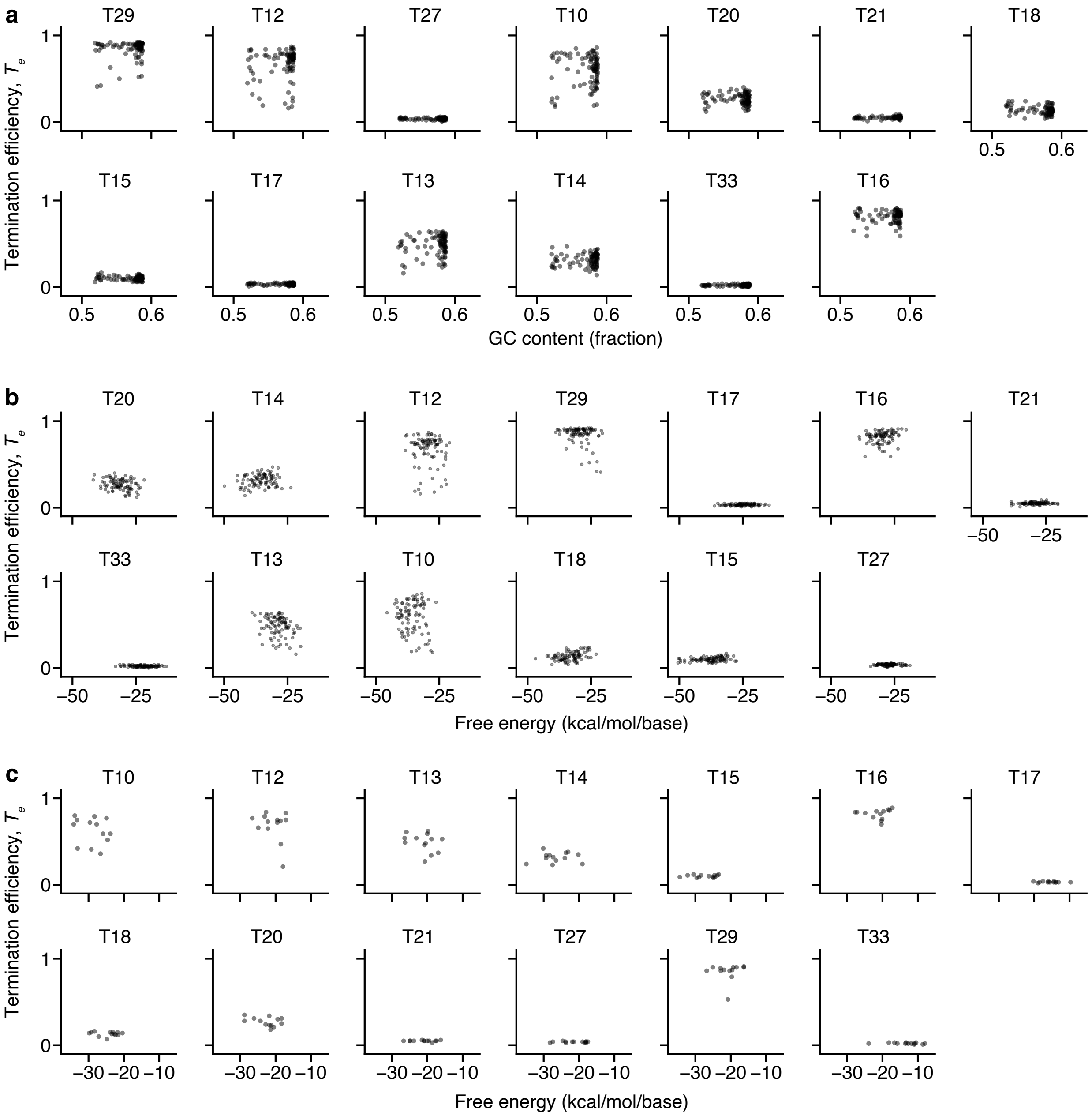

Supplementary Figure 8: Analysis of possible predictors of termination efficiency. (**a**) Scatter plot for each terminator showing T_e against percentage GC content of each design. Calculation based on 80 nt upstream of 3'-end of design. (**b**) Scatter plot for each terminator showing T_e against the thermodynamic minimum free energy of each design. Calculation using default settings, based on 120 nt upstream of 3'-end of the design. (**c**) Scatter plot for each valve showing T_e against the thermodynamic minimum free energy of each valve sequence. All folding energies calculated using RNAfold [5].

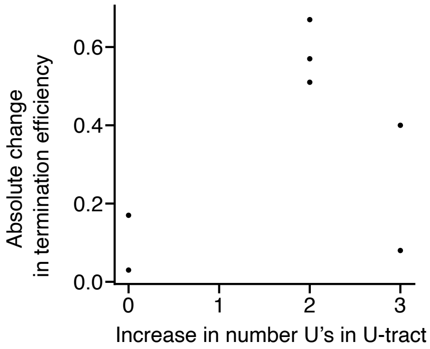

Supplementary Figure 9: Effect of U-tract changes on termination efficiency. Scatter plot showing how the absolute change in termination efficiency increases with an increasing number of U's in the U-tract. Each point corresponds to an individual terminator.

Supplementary Table 1: Oligonucleotide sequences

ID	Forward strand oligonucleotide sequence (5’–3’)	Reverse strand oligonucleotide sequence (5’–3’)	Library	Description
pT7	CTAATACGACTCACTATAGGGAGAG	CTAGCTCTCCCTATAGTGAGTCGTATTAGACGT	–	Promoter
S10	AATTCCTGTGTACCGGGAACCAGCCAGACTACACAGGGTAA	GCTCTTACCCTGTGTAGTCTGGCTGGTTCCCGGTACACAGG	L2	Spacer
S16	AATTCGTGCAGAGACAAGCGTTTGGGGCACCAGCACAGTAA	GCTCTTACTGTGCTGGTGCCCCAAACGCTTGTCTCTGCACG	L2	Spacer
S18	AATTCTTCAAAGCTACGAGCGCTAGAGATGTGAGACCCTAA	GCTCTTAGGGTCTCACATCTCTAGCGCTCGTAGCTTTGAAG	L2	Spacer
S19	AATTCCTAATTATGTCTCAAAAGCTCGAAGATTACACCTAA	GCTCTTAGGTGTAATCTTCGAGCTTTTGAGACATAATTAGG	L2	Spacer
S20	AATTCTTGTCGCTAAAGAAACCTTTCCCAATTAATACATAA	GCTCTTATGTATTAATTGGGAAAGGTTTCTTTAGCGACAAG	L2	Spacer
S21	AATTCGGAATCGCTGATCTACAGAACGGTCCTTATGGGTAA	GCTCTTACCCATAAGGACCGTTCTGTAGATCAGCGATTCCG	L2	Spacer
S22	AATTCATCACTCACACATCGCTCGAGATCGGTACGGGGTAA	GCTCTTACCCCGTACCGATCTCGAGCGATGTGTGAGTGATG	L2	Spacer
M10	GAGCTTTCTCCGAAGTGTAGTAAAAAAATAAAAA	GGCATTTTTATTTTTTTACTACACTTCGGAGAAA	L2	Modifier
M11	GAGCGATTACAGAAGCGTGGTATTTTTTATTTTT	GGCAAAAAATAAAAAATACCACGCTTCTGTAATC	L2	Modifier
M12	GAGCCAGGAACTTATCAATAGTCGCCCGAAAGGG	GGCACCCTTTCGGGCGACTATTGATAAGTTCCTG	L2	Modifier
M13	GAGCCCTATTTACCTCAGT	GGCAACTGAGGTAAATAGG	L2	Modifier
M14	GAGCTAGACAGTAATACCC	GGCAGGGTATTACTGTCTA	L2	Modifier
M15	GAGCCTATCTGGTGCTACA	GGCATGTAGCACCAGATAG	L2	Modifier
M16	GAGCTTATCGGTTACCAGA	GGCATCTGGTAACCGATAA	L2	Modifier
M17	GAGCGTATCCAGACTTATTGAGGTTTACGCACTA	GGCATAGTGCGTAAACCTCAATAAGTCTGGATAC	L2	Modifier
M18	GAGCATTCGCTGAGAGTTACACGATACTGACTAT	GGCAATAGTCAGTATCGTGTAACTCTCAGCGAAT	L2	Modifier
M19	GAGCTTGAAATCGGATACTTCCTGAACTGCGAAT	GGCAATTCGCAGTTCAGGAAGTATCCGATTTCAA	L2	Modifier
M20	GAGCATAGACTTTCGTGGATTATTACCTTACAACTGATAGGACGGACTC	GGCAGAGTCCGTCCTATCAGTTGTAAGGTAATAATCCACGAAAGTCTAT	L2	Modifier

M21	GAGCATAGCCGAGATTATCCACCAGCAACAGTTCGTTATTGTAGTGATT	GGCAAATCACTACAATAACGAACTGTTGCTGGTGGATAATCTCGGCTAT	L2	Modifier
M22	GAGCAAGGCGTGACTACAACCAATCTTCTATTCTGCGAGAGTAAAGTTT	GGCAAAACTTTACTCTCGCAGAATAGAAGATTGGTTGTAGTCACGCCTT	L2	Modifier
T10	TGCCGCTGATGCCAGAAAGGGTCCTGAATTTCAGGGCCCTTTTTTTACATGGATTGA	CTAGTCAATCCATGTAAAAAAAGGGCCCTGAAATTCAGGACCCTTTCTGGCATCAGC	L2	Terminator
T12	TGCCACTGATTTTTAAGGCGACTGATGAGTCGCCTTTTTTTTGTCTA	CTAGTAGACAAAAAAAAGGCGACTCATCAGTCGCCTTAAAAATCAGT	L2	Terminator
T13	TGCCAGTTAACCAAAAAGGGGGGATTTTATCTCCCCTTTAATTTTTCCTA	CTAGTAGGAAAAATTAAAGGGGAGATAAAATCCCCCCTTTTTGGTTAACT	L2	Terminator
T14	TGCCCGTGTTCCTGAACGCCCGCATATGCGGGCGTTTTGCTTTTTGA	CTAGTCAAAAAGCAAAACGCCCGCATATGCGGGCGTTCAGGAACACG	L2	Terminator
T15	TGCCTCTGAATGCGTGCCCATTCCTGACGGAATGGGCATTTCTGCGCAA	CTAGTTGCGCAGAAATGCCCATTCCGTCAGGAATGGGCACGCATTCAGA	L2	Terminator
T16	TGCCGTTATTAAATAGCCTGCCATCTGGCAGGCTTTTTTTATCGA	CTAGTCGATAAAAAAAGCCTGCCAGATGGCAGGCTATTTAATAAC	L2	Terminator
T17	TGCCCGTCTGCGTATGGAACGTGGTAACGGTTCTACTGAAGATTTA	CTAGTAAATCTTCAGTAGAACCGTTACCACGTTCCATACGCAGACG	L2	Terminator
T18	TGCCTACTTCTTACTCGCCCATCTGCAACGGATGGGCGAATTTATACCCA	CTAGTGGGTATAAATTCGCCCATCCGTTGCAGATGGGCGAGTAAGAAGTA	L2	Terminator
T20	TGCCCTGAAATATCCAGCGGATCAAGAAAATTCGTTGGATATTTTTTA	CTAGTAAAAAATATCCAACGAATTTTCTTGATCCGCTGGATATTTCAG	L2	Terminator
T21	TGCCAAACACGTAGGCCTGATAAGCGAAGCGCATCAGGCAGTTTTGCGTA	CTAGTACGCAAAACTGCCTGATGCGCTTCGCTTATCAGGCCTACGTGTTT	L2	Terminator
T27	TGCCTTTCAGCAAAAAACCCCTCAAGACCCGTTTAGAGGCCCCAAGGGGTTATGCTAGGA	CTAGTCCTAGCATAACCCCTTGGGGCCTCTAAACGGGTCTTGAGGGGTTTTTTGCTGAAA	L2	Terminator
T29	TGCCCAGAAATCATCCTTAGCGAAAGCTAAGGATTTTTTTTATCTGAAA	CTAGTTTCAGATAAAAAAAATCCTTAGCTTTCGCTAAGGATGATTTCTG	L2	Terminator
T33	TGCCCAGCGTTGAACCTACGACAGTCTCTTATTGACGAGTAAAGTGCTA	CTAGTAGCACTTTACTCGTCAATAAGAGACTGTCGTAGGTTCAACGCTG	L2	Terminator
S1	AATTCGACTTTCACGTGAACCTGTTCCCAATATAA	GCTCTTATATTGGGAACAGGTTCACGTGAAAGTCG	L1	Spacer
S2	AATTCAATGTGGAACTCTTCGCTCATGTAGAATAA	GCTCTTATTCTACATGAGCGAAGAGTTCCACATTG	L1	Spacer
S3	AATTCGGTGCAGCGGAGAAAAGATTTGCTACCTAA	GCTCTTAGGTAGCAAATCTTTTCTCCGCTGCACCG	L1	Spacer
S4	AATTCCTTGATATAAAACTTCCGGGAGTAGGATAA	GCTCTTATCCTACTCCCGGAAGTTTTATATCAAGG	L1	Spacer
S5	AATTCCAAGAACTCGTTTTCCTATATGGCGTCTAA	GCTCTTAGACGCCATATAGGAAAACGAGTTCTTGG	L1	Spacer
M1N	GAGCTTTCTCCGAAGTGTAGTAAATAAAGCGTCC	GGCAGGACGCTTTATTTACTACACTTCGGAGAAA	L1	Modifier

M1A	GAGCTTTCTCCGAAGTGTAGTAAATTTTATTTTT	GGCAAAAAATAAAATTTACTACACTTCGGAGAAA	L1	Modifier
M1U	GAGCTTTCTCCGAAGTGTAGTAAAAAAATAAAAA	GGCATTTTTATTTTTTTACTACACTTCGGAGAAA	L1	Modifier
M1S	GAGCTTTCTCCGAAGTGTAGTAAACCCGAAAGGG	GGCACCCTTTCGGGTTTACTACACTTCGGAGAAA	L1	Modifier
M1T	GAGCTTTCTCCGAAGTGTAGTAAA	GGCATTTACTACACTTCGGAGAAA	L1	Modifier
M1X	GAGCTTTCTCCGAAG	GGCACTTCGGAGAAA	L1	Modifier
M2N	GAGCAAGGACTTTCTCTACTGATTGTAAGACCGA	GGCATCGGTCTTACAATCAGTAGAGAAAGTCCTT	L1	Modifier
M2A	GAGCAAGGACTTTCTCTACTGATTTTTTATTTTT	GGCAAAAAATAAAAAATCAGTAGAGAAAGTCCTT	L1	Modifier
M2U	GAGCAAGGACTTTCTCTACTGATTAAAATAAAAA	GGCATTTTTATTTTAATCAGTAGAGAAAGTCCTT	L1	Modifier
M2S	GAGCAAGGACTTTCTCTACTGATTCCCGAAAGGG	GGCACCCTTTCGGGAATCAGTAGAGAAAGTCCTT	L1	Modifier
M2T	GAGCAAGGACTTTCTCTACTGATT	GGCAAATCAGTAGAGAAAGTCCTT	L1	Modifier
M2X	GAGCAAGGACTTTCT	GGCAAGAAAGTCCTT	L1	Modifier
M3N	GAGCCAGGAACTTATCAATAGTCGTTGTGACACT	GGCAAGTGTCACAACGACTATTGATAAGTTCCTG	L1	Modifier
M3A	GAGCCAGGAACTTATCAATAGTCGTTTTATTTTT	GGCAAAAAATAAAACGACTATTGATAAGTTCCTG	L1	Modifier
M3U	GAGCCAGGAACTTATCAATAGTCGAAAATAAAAA	GGCATTTTTATTTTCGACTATTGATAAGTTCCTG	L1	Modifier
M3S	GAGCCAGGAACTTATCAATAGTCGCCCGAAAGGG	GGCACCCTTTCGGGCGACTATTGATAAGTTCCTG	L1	Modifier
M3T	GAGCCAGGAACTTATCAATAGTCG	GGCACGACTATTGATAAGTTCCTG	L1	Modifier
M3X	GAGCCAGGAACTTAT	GGCAATAAGTTCCTG	L1	Modifier
T2	TGCCCGTAAAAACCCGCCGAAGCGGGTTTTTACGTAACA	CTAGTGTTACGTAAAAACCCGCTTCGGCGGGTTTTTACG	L1	Terminator
T3	TGCCAGTAAAAACCCGCCGAAGCGGGTTTTTACGTAACA	CTAGTGTTACGTAAAAACCCGCTTCGGCGGGTTTTTACT	L1	Terminator
T4	TGCCAAAAAAAACACCCTAACGGGTGTTTTTTTTTTTA	CTAGTAAAAAAAAAAAACACCCGTTAGGGTGTTTTTTTT	L1	Terminator
T5	TGCCAGAATTCAGTCAAAAGCCTCCGACCGGAGGCTTTTGACTATTACTACTAGA	CTAGTCTAGTAGTAATAGTCAAAAGCCTCCGGTCGGAGGCTTTTGACTGAATTCT	L1	Terminator
T6	TGCCAGAATTCAGCCCGCCTAATGAGCGGGCTTTTTTTTACTAA	CTAGTTAGTAAAAAAAAGCCCGCTCATTAGGCGGGCTGAATTCT	L1	Terminator

T7	TGCCAGAAAAGAGGCCTCCCGAAAGGGGGGCCTTTTTTCGTTTTA	CTAGTAAAACGAAAAAAGGCCCCCCTTTCGGGAGGCCTCTTTTCT	L1	Terminator
T50	AATTCTAAAAATATCCAACGAATTTTCTTGATCCGCTGGATATTTTTTTTCAGA	CTAGTCTGAAAAAAAATATCCAGCGGATCAAGAAAATTCGTTGGATATTTTTAG	L4	Terminator, +U-tract, T20
T51	AATTCTAAagttaaccaaAAAGGGGGGATTTTATCTCCCCTTTtttttcctA	CTAGTaggaaaaaAAAGGGGAGATAAAATCCCCCCTTTttggttaactTTAG	L4	Terminator, +U-tract, T13
T52	AATTCTAAcgtgttcctgAACGCCCGCATATGCGGGCGTTtttttttgA	CTAGTcaaaaaaaAACGCCCGCATATGCGGGCGTTcaggaacacgTTAG	L4	Terminator, +U-tract, T14
T53	AATTCTAAcgtctgcgtaTGGAACGTGGTAACGGTTCTATTTTTTTTctgaagatttA	CTAGTaaatcttcagAAAAAAAATAGAACCGTTACCACGTTCCAtacgcagacgTTAG	L4	Terminator, +U-tract, T17
T54	AATTCTAAtacttcttacTCGCCCATCTGCAACGGATGGGCGATTTTTTtttatacccA	CTAGTgggtataaaAAAAATCGCCCATCCGTTGCAGATGGGCGAgtaagaagtaTTAG	L4	Terminator, +U-tract, T18
T55	AATTCTAAaaacacgtagGCCTGATAAGCGAAGCGCATCAGGCTTTTttttgcgtA	CTAGTacgcaaaaAAAAGCCTGATGCGCTTCGCTTATCAGGCctacgtgtttTTAG	L4	Terminator, +U-tract, T21
T56	AATTCTAAtctgaatgcgTGCCCATTCCTGACGGAATGGGCATTTTTTtttctgcgcaA	CTAGTtgcgcagaaaAAAAATGCCCATTCCGTCAGGAATGGGCAcgcattcagaTTAG	L4	Terminator, +U-tract, T15
T57	AATTCTAACAGCGTTGAACCTACGACAGTCTCTTATTGACGAGTAAAGTGCTA	CTAGTAGCACTTTACTCGTCAATAAGAGACTGTCGTAGGTTCAACGCTGTTAG	L4	Terminator, Negative control, T33
T58	AATTCTAAAAATAGTTACCGAAAGTGTCCTGACCCAGTTGAGGCGTTTACTCA	CTAGTGAGTAAACGCCTCAACTGGGTCAGGACACTTTCGGTAACTATTTTTAG	L4	Terminator, Negative control, T34
T59	AATTCTAAAAGACCCCCGCACCGAAAGGTCCGGGGGTTTTTTTTA	CTAGTAAAAAAAACCCCCGGACCTTTCGGTGCGGGGGTCTTTTAG	L4	Terminator, *E. coli*, *ilvBN*
T60	AATTCTAAcaacaatgacAAGCGGTGGAGATCTTCTCTGCCGCTTttttttcatA	CTAGTatgaaaaaaAAGCGGCAGAGAAGATCTCCACCGCTTgtcattgttgTTAG	L4	Terminator, *E. coli*, ECK120015452
T61	AATTCTAAGTCAGTCGTCAGACGCCGGTTAATCCGGCGTTTTTTTTGACGCCCACA	CTAGTGTGGGCGTCAAAAAAAACGCCGGATTAACCGGCGTCTGACGACTGACTTAG	L4	Terminator, *E. coli*, ECK120051408
T62	AATTCTAAAAAAAAGTAACTAATGAGAAAAGCGCAGGGTGAAAGCCCTGCGCTTTTTCTTA	CTAGTAAGAAAAAGCGCAGGGCTTTCACCCTGCGCTTTTCTCATTAGTTACTTTTTTTAG	L4	Terminator, *B. subtilis*, *rpmF*
T63	AATTCTAAACTGAGTAATAGTATGGTTTTAAACGAGACCCCTGTGGGTCTCGTTTTTTGA	CTAGTCAAAAAACGAGACCCACAGGGGTCTCGTTTAAAACCATACTATTACTCAGTTTAG	L4	Terminator, *B. subtilis*, *tufA*
T64	AATTCTAAGAGGTGTAAGAAAAAAGCCAGAGCTTTGAAAAAGGTTCTGGCTTTTTTTCTA	CTAGTAGAAAAAAAGCCAGAACCTTTTTCAAAGCTCTGGCTTTTTTCTTACACCTCTTAG	L4	Terminator, *B. subtilis*, *rpmGA*
T65	AATTCTAACAGAGTAATCTGAAGCAACGTAAAAAAAACCCGCCCCGGCGGGTTTTTTTATA	CTAGTATAAAAAAACCCGCCGGGGCGGGTTTTTTTACGTTGCTTCAGATTACTCTGTTAG	L4	Terminator, *E. coli*, *rpl*

T66	AATTCTAAAAATGCTTGATTAAAAAGGCGCTACTCGGCATGGGGAAGCGCCTTTTTTATA	CTAGTATAAAAAAGGCGCTTCCCCATGCCGAGTAGCGCCTTTTTAATCAAGCATTTTTAG	L4	Terminator, *E. coli*, *fis*
T67	AATTCTAAGGCCGCATATCAGCTTAAAAAATGAACCATCGCCAACGGCGGTGGTTTTTTA	CTAGTAAAAAACCACCGCCGTTGGCGATGGTTCATTTTTTAAGCTGATATGCGGCCTTAG	L4	Terminator, *E. coli*, *sod*
T68	AATTCTAAATCTAAGCTCAATAAGAGGCTATCAGGCTTAACCGCTTGGTAGCCTTTTTGA	CTAGTCAAAAAGGCTACCAAGCGGTTAAGCCTGATAGCCTCTTATTGAGCTTAGATTTAG	L4	Terminator, *V. natriegens*, *groE*
T69	AATTCTAATTAGCTCTTAACTGAGTTGAAAAAGAGGCGGCTTTATAGTCGCCTTTTTTGA	CTAGTCAAAAAAGGCGACTATAAAGCCGCCTCTTTTTCAACTCAGTTAAGAGCTAATTAG	L4	Terminator, *V. natriegens*, *PN96_1*
T70	AATTCTAAGTAACGTTTTAAGTTAATAAGAAGCCCCGAGTTATGCTCGGGGCTTTTTGTA	CTAGTACAAAAAGCCCCGAGCATAACTCGGGGCTTCTTATTAACTTAAAACGTTACTTAG	L4	Terminator, *V. natriegens*, *PN96_2*
T71	AATTCTAAGATCGCTAGACTAAGAGACCCCGTCTTCCGAAAGGGAGGCGGGGTCTTTCTA	CTAGTAGAAAGACCCCGCCTCCCTTTCGGAAGACGGGGTCTCTTAGTCTAGCGATCTTAG	L4	Terminator, *C. crescentus*, *saA*
T72	AATTCTAAGCCTCTGACGATTCGAAAGCGCCGCCGGGTTTCGTCCCGGCGGCGCTTTTCA	CTAGTGAAAAGCGCCGCCGGGACGAAACCCGGCGGCGCTTTCGAATCGTCAGAGGCTTAG	L4	Terminator, *C. crescentus*, *CNA_1*
T73	AATTCTAACATAGCCCGTGAGCGAAACGCCCCGGAGGTCCGCCTCCGGGGCGTTTTTCTA	CTAGTAGAAAAACGCCCCGGAGGCGGACCTCCGGGGCGTTTCGCTCACGGGCTATGTTAG	L4	Terminator, *C. crescentus*, *CNA_2*
T74	AATTCTAATGGGGGGTATGGGGGGTATGGGGGGTATGGGGGGTATGGGGGGTATGGGGGA	CTAGTCCCCCATACCCCCCATACCCCCCATACCCCCCATACCCCCCATACCCCCCATTAG	L4	G-quadruplex
T75	AATTCTAATACTACGCTATCCACTCCGCCCCCTTGGGGCCTCTAAACGGGTCTTGAGGGGTTTTTTTTA	CTAGTAAAAAAAACCCCTCAAGACCCGTTTAGAGGCCCCAAGGGGGCGGAGTGGATAGCGTAGTATTAG	L4	Terminator, Tract variant, T-theta, poly-T
T76	AATTCTAAATCTAAGATATGAAGGGAATCCCCTTGGGGCCTCTAAACGGGTCTTGAGGGGAAAAAAAAA	CTAGTTTTTTTTTCCCCTCAAGACCCGTTTAGAGGCCCCAAGGGGATTCCCTTCATATCTTAGATTTAG	L4	Terminator, Tract variant, T-theta, poly-A
T77	AATTCTAAAGGCCAGATCTAGAAGCATGCCCCTTGGGGCCTCTAAACGGGTCTTGAGGGGCCCCCCCCA	CTAGTGGGGGGGGCCCCTCAAGACCCGTTTAGAGGCCCCAAGGGGCATGCTTCTAGATCTGGCCTTTAG	L4	Terminator, Tract variant, T-theta, poly-C
T78	AATTCTAATCGACGACGTAACCGGCCTTCCCCTTGGGGCCTCTAAACGGGTCTTGAGGGGGGGGGGGGA	CTAGTCCCCCCCCCCCCTCAAGACCCGTTTAGAGGCCCCAAGGGGAAGGCCGGTTACGTCGTCGATTAG	L4	Terminator, Tract variant, T-theta, poly-G
T79	AATTCTAATCTCCTGCATTCCTCGTACAGGGTCCTGAATTTCAGGGCCCTTTTTTTTA	CTAGTAAAAAAAAGGGCCCTGAAATTCAGGACCCTGTACGAGGAATGCAGGAGATTAG	L4	Terminator, Tract variant, T10, poly-T
T80	AATTCTAATCTCTTCTATCCCGTCAAACGGGTCCTGAATTTCAGGGCCCAAAAAAAAA	CTAGTTTTTTTTTGGGCCCTGAAATTCAGGACCCGTTTGACGGGATAGAAGAGATTAG	L4	Terminator, Tract variant, T10, poly-A
T81	AATTCTAAGGCCTAATATCTCACCCTAAGGGTCCTGAATTTCAGGGCCCCCCCCCCCA	CTAGTGGGGGGGGGGGCCCTGAAATTCAGGACCCTTAGGGTGAGATATTAGGCCTTAG	L4	Terminator, Tract variant, T10, poly-C

T82	AATTCTAATTAACAGAACACCAGAATCCGGGTCCTGAATTTCAGGGCCCGGGGGGGGA	CTAGTCCCCCCCCGGGCCCTGAAATTCAGGACCCGGATTCTGGTGTTCTGTTAATTAG	L4	Terminator, Tract variant, T10, poly-G
T83	AATTCTAATATGTTAAATACCGTTCGCCTCCTTAGCGAAAGCTAAGGATTTTTTTTA	CTAGTAAAAAAAATCCTTAGCTTTCGCTAAGGAGGCGAACGGTATTTAACATATTAG	L4	Terminator, Tract variant, T29, poly-T
T84	AATTCTAAACTGAACCACGAATACGCAATCCTTAGCGAAAGCTAAGGAAAAAAAAAA	CTAGTTTTTTTTTTCCTTAGCTTTCGCTAAGGATTGCGTATTCGTGGTTCAGTTTAG	L4	Terminator, Tract variant, T29, poly-A
T85	AATTCTAACACTCGTTTATCCTTAACTATCCTTAGCGAAAGCTAAGGACCCCCCCCA	CTAGTGGGGGGGGTCCTTAGCTTTCGCTAAGGATAGTTAAGGATAAACGAGTGTTAG	L4	Terminator, Tract variant, T29, poly-C
T86	AATTCTAAATTTCGTAATAACCTTTAGCTCCTTAGCGAAAGCTAAGGAGGGGGGGGA	CTAGTCCCCCCCCTCCTTAGCTTTCGCTAAGGAGCTAAAGGTTATTACGAAATTTAG	L4	Terminator, Tract variant, T29, poly-G
T87	AATTCTAATGTCTGAATGCCGTTATCTCGCCTGCCATCTGGCAGGCTTTTTTTTA	CTAGTAAAAAAAAGCCTGCCAGATGGCAGGCGAGATAACGGCATTCAGACATTAG	L4	Terminator, Tract variant, T16, poly-T
T88	AATTCTAAGAGCATACGAATAGAACATGGCCTGCCATCTGGCAGGCAAAAAAAAA	CTAGTTTTTTTTTGCCTGCCAGATGGCAGGCCATGTTCTATTCGTATGCTCTTAG	L4	Terminator, Tract variant, T16, poly-A
T89	AATTCTAAACGGGTAGAAGGATGACAACGCCTGCCATCTGGCAGGCCCCCCCCCA	CTAGTGGGGGGGGGCCTGCCAGATGGCAGGCGTTGTCATCCTTCTACCCGTTTAG	L4	Terminator, Tract variant, T16, poly-C
T90	AATTCTAAAAGTGTGAGGTCAAATAAAGGCCTGCCATCTGGCAGGCGGGGGGGGA	CTAGTCCCCCCCCGCCTGCCAGATGGCAGGCCTTTATTTGACCTCACACTTTTAG	L4	Terminator, Tract variant, T16, poly-G
T99	TGCCAACTAGCATAACCCCTTGGGGCCTCTAAACGGGTCTTGAGGGGTTTTTTGCA	CTAGTTGCAAAAAACCCCTCAAGACCCGTTTAGAGGCCCCAAGGGGTTATGCTAGTT	L4, L3	Terminator, T-theta
M50	GAGCTACTACGCTATCCACTCCGCTTCCCATCACGAGATCTTGAAATTC	GGCAGAATTTCAAGATCTCGTGATGGGAAGCGGAGTGGATAGCGTAGTA	L3	Modifier, T10 Loop interactor (near)
M51	GAGCATCTAAGATATGAAGGGAATTGGCACAACGACTGAACCAGGACCC	GGCAGGGTCCTGGTTCAGTCGTTGTGCCAATTCCCTTCATATCTTAGAT	L3	Modifier, T10 Stem1 interactor (near)
M52	GAGCAGGCCAGATCTAGAAGCATGCACGACAACCCTTTCACGGGCCCTG	GGCACAGGGCCCGTGAAAGGGTTGTCGTGCATGCTTCTAGATCTGGCCT	L3	Modifier, T10 Stem2 interactor (near)
M53	GAGCTCGACGACGTAACCGGCCTTCTCAACATTATACAAATCCAGATGG	GGCACCATCTGGATTTGTATAATGTTGAGAAGGCCGGTTACGTCGTCGA	L3	Modifier, T16 Loop interactor (near)
M54	GAGCTACTTTGTCTTCTCTACCACCTCTTGCCCGTATGCCTTGGCAGGC	GGCAGCCTGCCAAGGCATACGGGCAAGAGGTGGTAGAGAAGACAAAGTA	L3	Modifier, T16 Stem1 (near)
M55	GAGCGCATAAAGACGGGAGAAAGAGTTACAGCAAAGAGAAGGCCTGCCA	GGCATGGCAGGCCTTCTCTTTGCTGTAACTCTTTCTCCCGTCTTTATGC	L3	Modifier, T16 Stem2 (near)
M56	GAGCTAGTTACCCAACCGTGCGCATGTGATCCTGTTAAGATGCTTTCGC	GGCAGCGAAAGCATCTTAACAGGATCACATGCGCACGGTTGGGTAACTA	L3	Modifier, T29 Loop interactor (near)

M57	GAGCTCTCCTGCATTCCTCGTACATAGCTTAAACTTGATTTAAGGATGA	GGCATCATCCTTAAATCAAGTTTAAGCTATGTACGAGGAATGCAGGAGA	L3	Modifier, T29 Stem1 interactor (near)
M58	GAGCTCTCTTCTATCCCGTCAAACTAAGTACCAAACGTCATTCCTTAGC	GGCAGCTAAGGAATGACGTTTGGTACTTAGTTTGACGGGATAGAAGAGA	L3	Modifier, T29 Stem2 interactor (near)
M59	GAGCTGGTGCGTAGTAGACTTAACAAGATGTGATTTCGAAGCGTTTAGA	GGCATCTAAACGCTTCGAAATCACATCTTGTTAAGTCTACTACGCACCA	L3	Modifier, T-theta Loop interactor (near)
M60	GAGCGTGTAGGTATGTGTCGGTCGTTGTGGTGGTTTAGTGTCCAAGGGG	GGCACCCCTTGGACACTAAACCACCACAACGACCGACACATACCTACAC	L3	Modifier, T-theta Stem1 interactor (near)
M61	GAGCTAATTCTGAGCTAGACTATGATTCCCTCCAACAAATGCCCCTCAA	GGCATTGAGGGGCATTTGTTGGAGGGAATCATAGTCTAGCTCAGAATTA	L3	Modifier, T-theta Stem2 interactor (near)
M62	GAGCTGAAATTCGGCCTAATATCTCACCCTAAAGACTAATACTTCCCGC	GGCAGCGGGAAGTATTAGTCTTTAGGGTGAGATATTAGGCCGAATTTCA	L3	Modifier, T10 Loop interactor (far)
M63	GAGCCAGGACCCTTAACAGAACACCAGAATCCTAGCGAACCCAACCTCT	GGCAAGAGGTTGGGTTCGCTAGGATTCTGGTGTTCTGTTAAGGGTCCTG	L3	Modifier, T10 Stem1 interactor (far)
M64	GAGCGGGCCCTGGCCGAACGTCTAGCCCACCAAATGAAGCCTTAAGAGA	GGCATCTCTTAAGGCTTCATTTGGTGGGCTAGACGTTCGGCCAGGGCCC	L3	Modifier, T10 Stem2 interactor (far)
M65	GAGCCCAGATGGGAACCGCCATCTAACAGAAGTAAACATTACCCATCAG	GGCACTGATGGGTAATGTTTACTTCTGTTAGATGGCGGTTCCCATCTGG	L3	Modifier, T16 Loop interactor (far)
M66	GAGCTGGCAGGCTCCGCTGCACTCCCGTTAATCCCATATTATTCTTCAT	GGCAATGAAGAATAATATGGGATTAACGGGAGTGCAGCGGAGCCTGCCA	L3	Modifier, T16 Stem1 interactor (far)
M67	GAGCGCCTGCCATATGTTAAATACCGTTCGCCTGCTTAACCTACTTGAT	GGCAATCAAGTAGGTTAAGCAGGCGAACGGTATTTAACATATGGCAGGC	L3	Modifier, T16 Stem2 interactor (far)
M68	GAGCGCTTTCGCACTGAACCACGAATACGCAAATAACCCAGCTACCGAA	GGCATTCGGTAGCTGGGTTATTTGCGTATTCGTGGTTCAGTGCGAAAGC	L3	Modifier, T29 Loop interactor (far)
M69	GAGCAAGGATGACACTCGTTTATCCTTAACTATCTACCTTATCACTCTA	GGCATAGAGTGATAAGGTAGATAGTTAAGGATAAACGAGTGTCATCCTT	L3	Modifier, T29 Stem1 interactor (far)
M70	GAGCTCCTTAGCATTTCGTAATAACCTTTAGCATAGCATCACAGACTAC	GGCAGTAGTCTGTGATGCTATGCTAAAGGTTATTACGAAATGCTAAGGA	L3	Modifier, T29 Stem2 interactor (far)
M71	GAGCCGTTTAGATTAAGAGAGGAGATAGTCAATAACAGAACAAGAGCGT	GGCAACGCTCTTGTTCTGTTATTGACTATCTCCTCTCTTAATCTAAACG	L3	Modifier, T-theta Loop interactor (far)
M72	GAGCCCAAGGGGGCCGGATTCGAACATTCTACCTATTACACAGCTTTAA	GGCATTAAAGCTGTGTAATAGGTAGAATGTTCGAATCCGGCCCCCTTGG	L3	Modifier, T-theta Stem1 interactor (far)
M73	GAGCCCCCTCAAGGAGAGTTAATCGAAGAGAATCAGACACAAGGCGGAA	GGCATTCCGCCTTGTGTCTGATTCTCTTCGATTAACTCTCCTTGAGGGG	L3	Modifier, T-theta Stem2 interactor (far)

M74	GAGCCAGCCCAGAGTAGTATTTCTCCGAAGTGTAGTAAAAAAATAAAAA	GGCATTTTTATTTTTTTACTACACTTCGGAGAAATACTACTCTGGGCTG	L3	Modifier, M11 A-tract interactor (near)
M75	GAGCCGACGTGTTATCTAAGATTACAGAAGCGTGGTATTTTTTATTTTT	GGCAAAAAATAAAAAATACCACGCTTCTGTAATCTTAGATAACACGTCG	L3	Modifier, M11 T-tract interactor (near)
M76	GAGCAAAAAATAAAAAGGAGGAGCAGCATACTTACAAGACACGGGATAT	GGCAATATCCCGTGTCTTGTAAGTATGCTGCTCCTCCTTTTTATTTTTT	L3	Modifier, A-tract interactor (far)
M77	GAGCTTTTTTATTTTTTGTCTGAATGCCGTTATCTCTTCTCAATATGAA	GGCATTCATATTGAGAAGAGATAACGGCATTCAGACAAAAAATAAAAAA	L3	Modifier, T-tract interactor (far)
M78	GAGCGAATAGAACATGGAACAATCAGCACTGAGCGGCACTTCGGTGCCG	GGCACGGCACCGAAGTGCCGCTCAGTGCTGATTGTTCCATGTTCTATTC	L3	Modifier, Long HP TTCG (near)
M79	GAGCACGGGTAGAAGGATGACAACGGGAAGAGCGCACGGgagaCCGTGC	GGCAGCACGGtctcCCGTGCGCTCTTCCCGTTGTCATCCTTCTACCCGT	L3	Modifier, Long HP GAGA (near)
M80	GAGCAAGTGTGAGGTCAAATAAAGCAATAAGTCGGTGCCGAAAGGCACC	GGCAGGTGCCTTTCGGCACCGACTTATTGCTTTATTTGACCTCACACTT	L3	Modifier, Long HP GAAA (near)
M81	GAGCGTAGTAGATAAGGTGGATGTGGGACGAGCAGTGGGGCGTTCGCGC	GGCAGCGCGAACGCCCCACTGCTCGTCCCACATCCACCTTATCTACTAC	L3	Modifier, Short HP TTCG (near)
M82	GAGCGATTAGGAAGATTAGGCACATTACAGACTGGAACGCCCGAAAGGG	GGCACCCTTTCGGGCGTTCCAGTCTGTAATGTGCCTAATCTTCCTAATC	L3	Modifier, M12 Short HP GAAA (near)
M83	GAGCGGCTAAAGAGAAAGATTAACGTTCCACACACGCGACGCGAGAGCG	GGCACGCTCTCGCGTCGCGTGTGTGGAACGTTAATCTTTCTCTTTAGCC	L3	Modifier, Short HP GAGA (near)
M84	GAGCCGGCACTTCGGTGCCGTTTCCTTATTGCCCTCGCCCGTCGCCCGA	GGCATCGGGCGACGGGCGAGGGCAATAAGGAAACGGCACCGAAGTGCCG	L3	Modifier, Long HP TTCG (far)
M85	GAGCGCACGGgagaCCGTGCATAGAATAGTAACACAGCGCGCAAGGAAC	GGCAGTTCCTTGCGCGCTGTGTTACTATTCTATGCACGGtctcCCGTGC	L3	Modifier, Long HP GAGA (far)
M86	GAGCGGTGCCGAAAGGCACCATTAGTACACTCACGACCCGATCCCTTAG	GGCACTAAGGGATCGGGTCGTGAGTGTACTAATGGTGCCTTTCGGCACC	L3	Modifier, Long HP GAAA (far)
M87	GAGCGCGTTCGCGCAAGACCAATTTCAAAGCGGCGTCCTTATTTACCAT	GGCAATGGTAAATAAGGACGCCGCTTTGAAATTGGTCTTGCGCGAACGC	L3	Modifier, Short HP TTCG (far)
M88	GAGCTCGCTGCCCAACAACTTACTCTCGAATATGACACTCCCGAAAGGG	GGCACCCTTTCGGGAGTGTCATATTCGAGAGTAAGTTGTTGGGCAGCGA	L3	Modifier, M12 Short HP GAAA (far)
M89	GAGCCGCGAGAGCGGCAAGTTAAGACCGTACATCACTTCAAGAGAGCAC	GGCAGTGCTCTCTTGAAGTGATGTACGGTCTTAACTTGCCGCTCTCGCG	L3	Modifier, Short HP GAGA (far)
M90	GAGCCCGTTGTTGTTTGATCGGCAGATCTGAGCCTGGGAGCTCTCTGCC	GGCAGGCAGAGAGCTCCCAGGCTCAGATCTGCCGATCAAACAACAACGG	L3	Modifier, Structure Pseudoknot1

M91	GAGCCCTGTGCCGAGGGCGCAGTGGGCTAGCGCCACTCAAAAGGCCCAT	GGCAATGGGCCTTTTGAGTGGCGCTAGCCCACTGCGCCCTCGGCACAGG	L3	Modifier, Structure Pseudoknot2
S10	AATTCCTGTGTACCGGGAACCAGCCAGACTACACAGGGTAA	GCTCTTACCCTGTGTAGTCTGGCTGGTTCCCGGTACACAGG	L3	Spacer
S16	AATTCGTGCAGAGACAAGCGTTTGGGGCACCAGCACAGTAA	GCTCTTACTGTGCTGGTGCCCCAAACGCTTGTCTCTGCACG	L3	Spacer
S18	AATTCTTCAAAGCTACGAGCGCTAGAGATGTGAGACCCTAA	GCTCTTAGGGTCTCACATCTCTAGCGCTCGTAGCTTTGAAG	L3	Spacer
S19	AATTCCTAATTATGTCTCAAAAGCTCGAAGATTACACCTAA	GCTCTTAGGTGTAATCTTCGAGCTTTTGAGACATAATTAGG	L3	Spacer
S20	AATTCTTGTCGCTAAAGAAACCTTTCCCAATTAATACATAA	GCTCTTATGTATTAATTGGGAAAGGTTTCTTTAGCGACAAG	L3	Spacer
S21	AATTCGGAATCGCTGATCTACAGAACGGTCCTTATGGGTAA	GCTCTTACCCATAAGGACCGTTCTGTAGATCAGCGATTCCG	L3	Spacer
S22	AATTCATCACTCACACATCGCTCGAGATCGGTACGGGGTAA	GCTCTTACCCCGTACCGATCTCGAGCGATGTGTGAGTGATG	L3	Spacer
T10	TGCCGCTGATGCCAGAAAGGGTCCTGAATTTCAGGGCCCTTTTTTTACATGGATTGA	CTAGTCAATCCATGTAAAAAAAGGGCCCTGAAATTCAGGACCCTTTCTGGCATCAGC	L3	Terminator
T16	TGCCGTTATTAAATAGCCTGCCATCTGGCAGGCTTTTTTTATCGA	CTAGTCGATAAAAAAAGCCTGCCAGATGGCAGGCTATTTAATAAC	L3	Terminator
T29	TGCCCAGAAATCATCCTTAGCGAAAGCTAAGGATTTTTTTTATCTGAAA	CTAGTTTCAGATAAAAAAAATCCTTAGCTTTCGCTAAGGATGATTTCTG	L3	Terminator
M20	GAGCATAGACTTTCGTGGATTATTACCTTACAACTGATAGGACGGACTC	GGCAGAGTCCGTCCTATCAGTTGTAAGGTAATAATCCACGAAAGTCTAT	L3	Modifier (reference)
M21	GAGCATAGCCGAGATTATCCACCAGCAACAGTTCGTTATTGTAGTGATT	GGCAAATCACTACAATAACGAACTGTTGCTGGTGGATAATCTCGGCTAT	L3	Modifier (reference)
M22	GAGCAAGGCGTGACTACAACCAATCTTCTATTCTGCGAGAGTAAAGTTT	GGCAAAACTTTACTCTCGCAGAATAGAAGATTGGTTGTAGTCACGCCTT	L3	Modifier (reference)

Supplementary Table 2: Designed libraries

Library	Description	Figures	Parts used for assembly or specific designs	Total designs
L1	Initial set to test methodology	SI2	S1, S2, S3, S4, S5<br><br>M1N, M1A, M1U, M1S, M1T, M1X, M2N, M2A, M2U, M2S, M2T, M2X, M3N, M3A, M3U, M3S, M3T, M3X<br><br>T2, T3, T4, T5, T6, T7	540
L2	Random library to explore all design parameters	1, 2, 3	S10, S16, S18, S19, S20, S21, S22<br><br>M10, M11, M12, M13, M14, M15, M16, M17, M18, M19, M20, M21, M22<br><br>T10, T12, T13, T14, T15, T16, T17, T18, T20, T21, T27, T29, T33	1183
L3	Library focused on modifier design principles	4	S10, S16, S18, S19, S20, S21, S22<br><br>M50, M51, M52, M53, M54, M55, M56, M57, M58, M59, M60, M61, M62, M63, M64, M65, M66, M67, M68, M69, M70, M71, M72, M73, M74, , M76, M77, M78, M79, M80, M81, M82, M83, M84, M85, M86, M87, M88, M89, M90, M91<br><br>T10, T16, T29, T99	1260
L4	Library focused on terminator design principles	5, 6C	T50, T51, T52, T53, T54, T55, T56, T57, T58, T59, T60, T61, T62, T63, T64, T65, T66, T67, T68, T69, T70, T71, T72, T73, T74, T75*, T76, T77, T78, T79, T80, T81, T82, T83, T84, T85, T86, T87, T88, T89, T90	41
L5	CRISPR gRNA arrays	6A, 6B	S20-M22-T29, S21-M10-T16, S10-M17-T20, S16-M12-T13, S18-M18-T33, S19-M19-T34	6

* T75 also referred to as T99U

Supplementary References

[1] Garalde, D. R. *et al.* Highly parallel direct RNA sequencing on an array of nanopores. *Nature Methods* **15**, 201–206 (2018).

[2] Depledge, D. P. *et al.* Direct RNA sequencing on nanopore arrays redefines the transcriptional complexity of a viral pathogen. *Nature Communications* **10**, 754 (2019).

[3] Li, H. *et al.* The Sequence Alignment/Map format and SAMtools. *Bioinformatics* **25**, 2078–2079 (2009).

[4] Patrick, Wayne M., Andrew E. Firth, and Jonathan M. Blackburn. User-Friendly Algorithms for Estimating Completeness and Diversity in Randomized Protein-Encoding Libraries. *Protein Engineering* **16**, 451–57 (2003).

[5] Lorenz, Ronny, Stephan H. Bernhart, Christian Hoener zu Siederdissen, Hakim Tafer, C. Peter Stadler and Ivo L. Hofacker. ViennaRNA Package 2.0, *Algorithms for Molecular Biology*: **6**, 26 (2011).
