# Supplementary material for: Massively parallel characterization of engineered transcript isoforms using direct RNA sequencing
Source: Nat Commun. 2022 Jan 21;13:434. doi: 10.1038/s41467-022-28074-5 (PMC8783025; doi:10.1038/s41467-022-28074-5)
Supplement: Supplementary file 2 — Description of Additional Supplementary Files [file 41467_2022_28074_MOESM2_ESM.pdf]

Title: Supplementary Data 1:
Description: Annotated plasmid sequences for all transcriptional valve libraries.

Title: Supplementary Data 2:
Description: Termination efficiency of studied transcriptional valves.
