# Supplementary material for: Massively parallel characterization of engineered transcript isoforms using direct RNA sequencing
Source: Nat Commun. 2022 Jan 21;13:434. doi: 10.1038/s41467-022-28074-5 (PMC8783025; doi:10.1038/s41467-022-28074-5)
Supplement: Supplementary file 5 — Reporting Summary [file 41467_2022_28074_MOESM5_ESM.pdf]

nature research

Corresponding author(s): Thomas Gorochowski

Last updated by author(s): Jan 6, 2022

Reporting Summary

Nature Research wishes to improve the reproducibility of the work that we publish. This form provides structure for consistency and transparency in reporting. For further information on Nature Research policies, see our Editorial Policies and the Editorial Policy Checklist.

Statistics

For all statistical analyses, confirm that the following items are present in the figure legend, table legend, main text, or Methods section.

n/a	Confirmed	
☐	☒	The exact sample size (n) for each experimental group/condition, given as a discrete number and unit of measurement
☐	☒	A statement on whether measurements were taken from distinct samples or whether the same sample was measured repeatedly
☒	☐	The statistical test(s) used AND whether they are one- or two-sided<br>*Only common tests should be described solely by name; describe more complex techniques in the Methods section.*
☒	☐	A description of all covariates tested
☒	☐	A description of any assumptions or corrections, such as tests of normality and adjustment for multiple comparisons
☐	☒	A full description of the statistical parameters including central tendency (e.g. means) or other basic estimates (e.g. regression coefficient) AND variation (e.g. standard deviation) or associated estimates of uncertainty (e.g. confidence intervals)
☒	☐	For null hypothesis testing, the test statistic (e.g. F, t, r) with confidence intervals, effect sizes, degrees of freedom and P value noted<br>*Give P values as exact values whenever suitable.*
☒	☐	For Bayesian analysis, information on the choice of priors and Markov chain Monte Carlo settings
☒	☐	For hierarchical and complex designs, identification of the appropriate level for tests and full reporting of outcomes
☐	☒	Estimates of effect sizes (e.g. Cohen's d, Pearson's r), indicating how they were calculated

Our web collection on statistics for biologists contains articles on many of the points above.

Software and code

Policy information about availability of computer code

Data collection	MinKNOW version 20.06.5 was used to collect all Direct RNA and DNA sequencing data.
Data analysis	Sequencing data was analysed using guppy version 3.1.5, BLASTN version 2.2.31, minimap2 version 2.17, Racon version 1.4, SAMtools version 1.11 and pySAM version 0.16. Analysis of all other data (e.g. calculation of statistics) and plotting was performed with RNAInverse version 2.5, R2oDNA Designer version 1.0, NullSeq version 1.0, Vienna RNAfold version 2.4.16, Multiple Alignment using Fast Fourier Transform (MAFFT) version 7, NumPy version 1.18.5, SciPy version 1.5.4, Pandas version 1.0.5, matplotlib version 3.2.2, and DNAplotlib version 1.0 using Python version 3.5.

For manuscripts utilizing custom algorithms or software that are central to the research but not yet described in published literature, software must be made available to editors and reviewers. We strongly encourage code deposition in a community repository (e.g. GitHub). See the Nature Research guidelines for submitting code & software for further information.

Data

Policy information about availability of data

All manuscripts must include a data availability statement. This statement should provide the following information, where applicable:

- Accession codes, unique identifiers, or web links for publicly available datasets
- A list of figures that have associated raw data
- A description of any restrictions on data availability

The sequencing data generated by this study have been deposited in the OSF database under DOI: 10.17605/OSF.IO/DUSPK. Plasmid sequences for all libraries in FASTA and GFF formats, are available in Supplementary Data 1. The termination efficiency of all transcriptional valves is summarized in Supplementary Data 2. Source data are provided with this paper for Figures 2–6.

Field-specific reporting

Please select the one below that is the best fit for your research. If you are not sure, read the appropriate sections before making your selection.

[x] Life sciences   [ ] Behavioural & social sciences   [ ] Ecological, evolutionary & environmental sciences

For a reference copy of the document with all sections, see nature.com/documents/nr-reporting-summary-flat.pdf

Life sciences study design

All studies must disclose on these points even when the disclosure is negative.

Sample size	Sample sizes were chosen to match typical practices in the field (e.g. 10s to 100s of DNA constructs characterised).
Data exclusions	No data were excluded from the analyses.
Replication	Direct RNA sequencing experiments were performed in biological duplicate and a strong correlation (R squared = 0.99) was found for measurements of the termination efficiency of each transcriptional valve between these replicates.
Randomization	Randomization is not relevant for this study as all DNA constructs (transcriptional valves) are pooled and simultaneously characterized.
Blinding	Blinding is not relevant for this study as all DNA constructs (transcriptional valves) are pooled and simultaneously characterized.

Reporting for specific materials, systems and methods

We require information from authors about some types of materials, experimental systems and methods used in many studies. Here, indicate whether each material, system or method listed is relevant to your study. If you are not sure if a list item applies to your research, read the appropriate section before selecting a response.

Materials & experimental systems

n/a	Involved in the study	
[x]	[ ]	Antibodies
[x]	[ ]	Eukaryotic cell lines
[x]	[ ]	Palaeontology and archaeology
[x]	[ ]	Animals and other organisms
[x]	[ ]	Human research participants
[x]	[ ]	Clinical data
[x]	[ ]	Dual use research of concern

Methods

n/a	Involved in the study	
[x]	[ ]	ChIP-seq
[x]	[ ]	Flow cytometry
[x]	[ ]	MRI-based neuroimaging
